# Dimerization in TMPA-Based Copper(I) Complexes: Implications for Redox Kinetics and Thermodynamics

**DOI:** 10.1021/acs.inorgchem.5c01099

**Published:** 2025-06-16

**Authors:** Marcos Tapia, Shyam K. Pahari, Sandip Das, Firoz Shah Tuglak Khan, Shabnam Hematian

**Affiliations:** Department of Chemistry and Biochemistry, 14616 University of North Carolina at Greensboro, Greensboro, North Carolina 27402, United States

## Abstract

Copper complexes of tris­(2-pyridylmethyl)­amine (TPA, TMPA, or TPMA) are widely studied for their redox activity and catalytic versatility. This study investigates solvent-dependent speciation of copper­(I)-TMPA complexes, revealing a monomer–dimer equilibrium in solution. Using electrochemical analysis, variable-temperature NMR spectroscopy, and X-ray crystallography, we identify an embraced dimer with a lower reduction potential and faster electron transfer than the monomer. Notably, while the monomer displays strong solvent dependence, the dimer’s redox properties remain largely invariant, reflecting its intrinsic stability.

## Graphical abstract

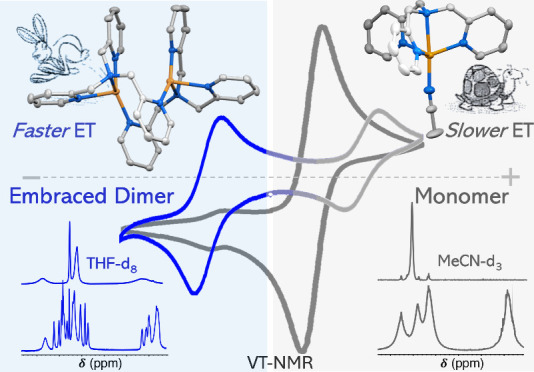

Tris­(2-pyridylmethyl)­amine (TPA, TMPA, or TPMA) is a versatile neutral tripodal nitrogen containing chelating agent widely used in the coordination chemistry with metals such as zinc,^1,2^ silver,^3^ mercury,^5^ iron,^6,7^ and copper.^4,8–11^ Therefore, TMPA and its derivatives have broad applications in fields including molecular catalysis,^12–16^ atom-transfer radical addition (ATRA)^17^ and polymerization (ATRP),^18^ supramolecular chemistry,^19^ materials science (e.g., metal–organic frameworks, MOFs),^20,21^ and metal ion chelation in biological systems.^1,2,8,9^

The coordination chemistry of TMPA has been extensively studied, with particular focus on its copper­(I) and copper­(II) complexes due to their favorable redox properties and catalytic activities. These complexes are known to activate small molecules such as dioxygen (O_2_),^10,11,22,23^ organohalides,^24^ and various nitrogen oxides (NO_*x*_),^25–27^ including nitric oxide, nitrite, and hyponitrite, highlighting their relevance in catalytic transformations and as models for enzymatic processes.^28,29^

Copper­(I), a d^10^ metal ion with no ligand field stabilization, supports a wide range of geometries, tetrahedral, square planar, trigonal-planar, or linear, depending on the ligand environments. This geometric flexibility gives rise to dynamic solution behavior. Despite TMPA’s inherent 3-fold symmetry, it can accommodate diverse coordination geometries.^30^ Notably, some Ag­(I) and Cu­(I) complexes are known to form dimers and oligomers, as evidenced by NMR spectroscopy and X-ray crystallography.^3,31–40^ While TMPA-based copper­(I) complexes are widely used, especially in studies of copper-O_2_ reactivity,^10,11,37,41,42^ their solution-phase speciation across varying solvents and temperatures has generally been presumed to be monomeric.

In a recent study from our laboratory,^43^ the oxygenation of copper­(I) complexes of TMPA and two fluorine-tagged derivatives, F_2_TMPA ≡ 1-(3-fluoropyridin-2-yl)-N-((3-fluoropyridin-2-yl)­methyl)-N-(pyridin-2-ylmethyl)­methanamine and MeTFE-TMPA ≡ 1-(3-methyl-4-(2,2,2-trifluoroethoxy)­pyridin-2-yl)-N,N-bis­(pyridin-2-ylmethyl)­methanamine, was examined using ^1^H and ^19^F NMR spectroscopies, alongside variable-temperature UV–vis measurements, in tetrahydro­furan (THF) and 2-methyltetrahydrofuran (MeTHF). The observed reactivity toward O_2_ in these solvents was faster than in strongly coordinating solvents, like acetonitrile (MeCN).^43,44^ Notably, Karlin and co-workers similarly proposed dimer involvement in acetone to explain O_2_ reactivity.^45^ While dimerization of copper­(I) complexes (Scheme 1) has been reported,^33,34^ its impact on redox behavior, particularly electron transfer kinetics and thermodynamics, remains unexplored.

**Scheme 1:**
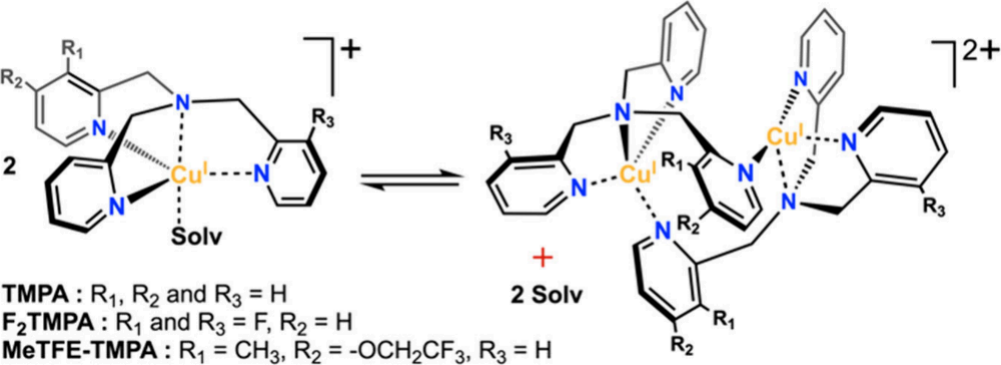
Proposed Dimerization Process

To address this longstanding ambiguity, we systematically investigate how dimerization affects the redox behavior of TMPA-based cuprous complexes across various organic solvents. Electrochemical analysis, variable-temperature NMR, and X-ray crystallography reveal that formation of embraced dimers, [(tmpa)_2_Cu^I^_2_]^2+^, significantly influences reduction potentials and electron transfer rates, offering new insights into their coordination chemistry and reactivity.

Alongside the parent TMPA, we examined one electron-rich and one electron-deficient fluorine-tagged derivative to enable ^19^F NMR spectroscopic analysis. Cyclic voltammetry (CV) in various solvents was used to probe solution speciation and dynamics of cuprous [(L)­Cu^I^]­[B­(C_6_F_5_)_4_] complexes (L = F_2_TMPA, TMPA, or MeTFE-TMPA), evaluating how different ligand environments, solvent coordination, and solvation properties impact the monomer–dimer equilibrium, complex stability, and redox properties.

CV revealed two distinct quasi-reversible redox events (Figure 1). The redox wave with a midpoint potential (*E*_*1/2*_) ranging from −140 to 410 mV vs Ag/AgCl corresponds to monomeric TMPA-based copper complexes. In contrast, the lower-potential peak (centered around −250 mV vs Ag/AgCl) is attributed to the embraced dimeric form, which undergoes a simultaneous two-electron transfer from both copper centers. This assignment is supported by the anodic-to-cathodic peak separation (Δ*E*_*p*_) value, which is nearly half that of the monomeric event (i.e., 36–97 mV vs 74–304 mV; Figure S3), consistent with Nernstian behavior for reversible multielectron processes.^46^ The two-electron reduction at a single potential observed for the embraced dimer suggests minimal electronic coupling between copper centers—an uncommon feature previously reported only in dicopper complexes with covalently fused ligands.^47–49^

**Figure 1:**
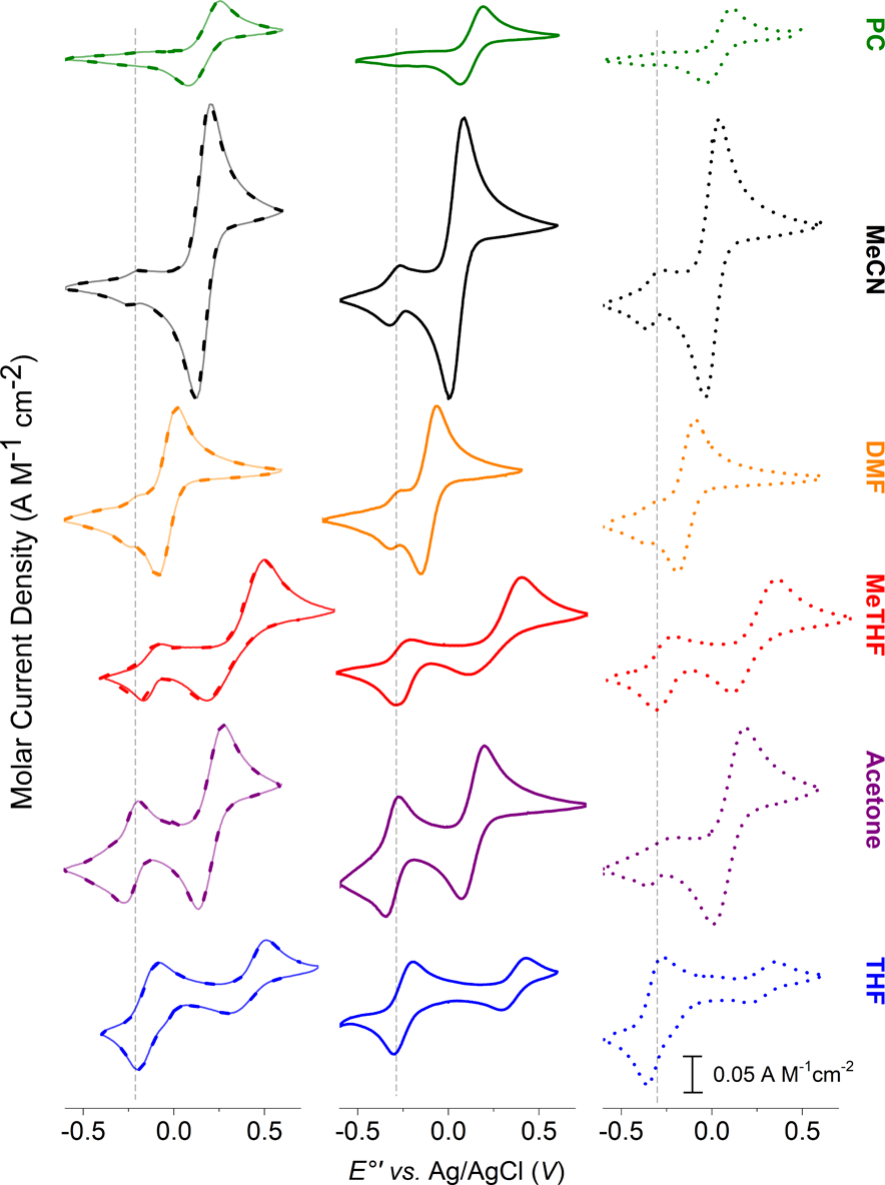
Cyclic voltammograms of [(F_2_tmpa)­Cu^I^]^+^ (left), [(tmpa)­Cu^I^]^+^ (middle), and [(MeTFE-tmpa)­Cu^I^]^+^ (right), demonstrating the solvent-dependent monomer–dimer equilibria. Peaks near −0.25 V correspond to the dimeric species.

Further evidence for this assignment comes from concentration-dependent studies, where the dimer-to-monomer ratio significantly increased at higher concentrations (Figure 2), confirming a dynamic equilibrium. Full data for *E*_*1/2*_, Δ*E*_*p*_, anodic to cathodic peak current ratios (*i*_*pa*_/*i*_*pc*_), diffusion coefficients (*D*_*0*_), heterogeneous electron-transfer rate constants (*k* ^*0*^), and simulated voltammograms for both species in each solvent are provided in Tables S2–S4, S7, and Figure S37. The Cu­(II)-dimer was modeled as a transient species—reversible on the CV time scale (*t*_*1/2*_ ≈ 147 s; Figure S30) but unstable in bulk, undergoing irreversible dissociation to monomers (Figure S36). Monomer/dimer ratios were estimated by peak integration, validated through chronoamperometry (Figure S2), scan-rate analysis, and digital simulations capturing the dynamic equilibrium (see the Supporting Information).

**Figure 2:**
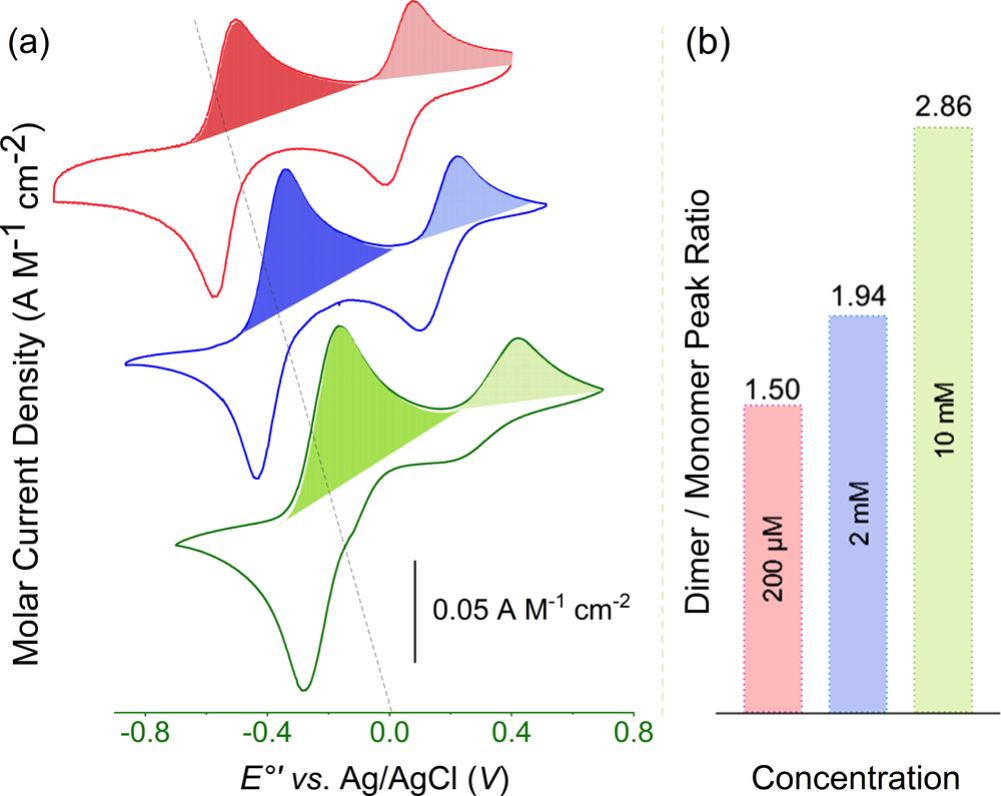
(a) Cyclic voltammograms of [(tmpa)­Cu^I^]­[B­(C_6_F_5_)_4_] in THF showing concentration-dependent monomer–dimer equilibrium. (b) Dimer-to-monomer peak area ratios increase with complex concentration.

Remarkably, the *E*_*1/2*_ values of the dimeric species remain almost unchanged across solvents (Figure S4), indicating minimal solvent influence due to shielding of the copper centers within the embraced structure. In contrast, monomeric complexes show marked solvent-dependent shifts in reduction potentials, correlating with solvent polarity and coordination ability (Table S5 and Figure S4). Polar solvents such as propylene carbonate (PC), MeCN, and dimethylformamide (DMF) appear to favor monomer formation, while lower-polarity solvents such as acetone, THF, and MeTHF promote dimerization (Figure 1 and Table S5). These trends align with our NMR studies (vide infra), highlighting solvent polarity (i.e., dipole moment and dielectric constant) as a key driver of the monomer–dimer equilibrium. The relevant solvent parameters are listed in Table S5.

Both dimeric and monomeric forms of all three TMPA-based copper­(I) complexes exhibit anodic/cathodic peak current ratios between 0.8 and 1.3 across all solvents, except for the parent complex in MeTHF, which shows values of 0.5 (dimer) and 1.6 (monomer) (Table S2).

Our Randles–Sevcik analysis confirmed, in all cases, diffusion-controlled redox behavior in all cases, with *D*_*0*_ values ranging from ∼0.3 to 13.6 × 10^–6^ cm^2^·s^–1^ (Figures S6–S23). As expected, all redox species diffused fastest in MeCN, consistent with its high polarity and low viscosity, which minimize analyte-electrolyte ion pairing and the solution resistance (Table S3 and Figure S31).

Additionally, the heterogeneous electron transfer rate constants (*k* ^*0*^ *)* for both monomeric and dimeric species were estimated using the Nicholson method, ranging from 0.3 to 34 × 10^–3^ cm·s^–1^ across solvents (Table S4 and Figures S24–S29, S32). The parent complex generally exhibited faster electron transfer than its fluorine-tagged analogues, and dimers consistently showed higher *k* ^*0*^ values than their corresponding monomers across all solvents, likely due to lower reorganization energies for the Cu^II/I^ couple. While *k* ^*0*^ does not directly dictate O_2_ reactivity, given the inner-sphere nature of O_2_ binding, these kinetic parameters, combined with the more favorable reduction potentials and lower coordination numbers of the dimers, may explain their enhanced reactivity in dimer-favoring solvents. These findings align with previously reported O_2_ reactivity trends^33,43,45^ and underscore the interplay between outer- and inner-sphere processes in redox chemistry. More broadly, they also offer a framework for tuning outer-sphere electron transfer in applications where copper speciation and solvent effects are critical, such as electrocatalysis, redox mediation, and redox flow batteries.

The roles of the electrolyte concentration, sample age, and counteranions, such as *tetrakis*(pentafluorophenyl)­borate ([B­(C_6_F_5_)_4_]^−^) and hexafluorophosphate ([PF_6_]^−^), in altering the monomer–dimer equilibrium were also briefly investigated. While electrolyte concentration had no apparent effect in MeCN, it influenced dimerization in lower polarity solvents such as acetone or THF (Figure S33). Time-dependent studies revealed no speciation changes in MeCN, but a gradual shift toward dimer formation in acetone and THF (Figures S33 and S34). Interestingly, the choice of the counteranion affected both solubility and speciation of these complexes in organic solvents. For example, even in MeCN, monomer predominance increased when the electrolyte anion was changed from the weakly ion-pairing [B­(C_6_F_5_)_4_]^−^ to the more traditional [PF_6_]^−^ (Figure S35). This suggests that the dimerization equilibrium is also sensitive to ion-pairing effects, which may explain why dimer formation was previously overlooked in electrochemical studies.^11,15,16,29,40,41,50^ Accordingly, solvent- and ion-dependent speciation, especially in media like DMF, acetone, or THF should not be underestimated.^16,22,42,51^

Of additional note is that, while recent studies^52,53^ on dipicolylaniline-supported chloro-bound copper complexes have attributed redox-active species to two monomeric conformers—arising from intrinsic conformational flexibility—and a bis­(μ-Cl) dimer at higher concentrations (≫1.5 mM), our study reveals a dynamic monomer–dimer equilibrium for TMPA-supported copper­(I) complexes. This embraced dimer forms even at relatively low concentrations (∼0.2 mM) and is dictated by the solution environment rather than halide bridging.

We further investigated the solution speciation using variable-temperature NMR spectroscopy. The ^1^H NMR spectra of the three complexes were collected in deuterated MeCN, DMF, acetone, or THF down to −80 °C. All three complexes exhibited distinct behavior in strongly coordinating MeCN compared to other solvents. In MeCN-*d*_*3*_, the spectra of [(tmpa)­Cu^I^]­[B­(C_6_F_5_)_4_] displayed a single broad peak near 4 ppm across all temperatures (Figure 3a), corresponding to the protons of three symmetry-equivalent methylene groups that link the central amine nitrogen to the pyridine rings. This pattern is consistent with a monomeric, solvent-bound structure [(tmpa)­Cu^I^(MeCN)]^+^, as reported in prior X-ray studies.^34^ The pronounced line broadening at higher temperatures is attributed to dynamic exchange processes, such as ligand and/or solvent dissociation/reassociation, which are typical for copper­(I) complexes.^45,54,55^

**Figure 3:**
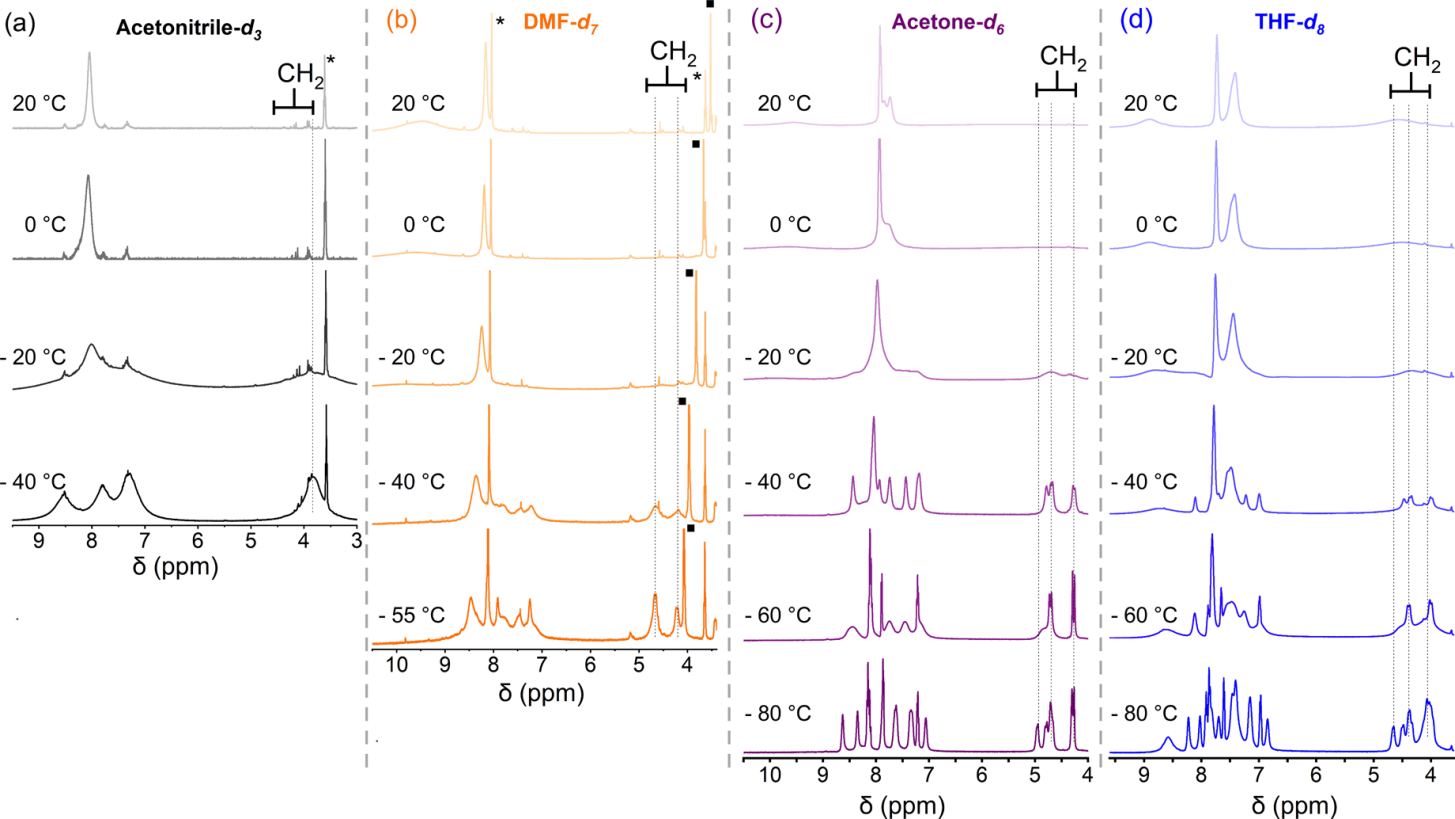
Variable-temperature ^1^H NMR spectra of [(tmpa)­Cu^I^]­[B­(C_6_F_5_)_4_] show well-resolved signals at low temperature in DMF-*d*_*7*_, acetone-*d*_*6*_, and THF-*d*_*8*_, consistent with a lower-symmetry dimer. * and ■ denote solvent residual and H_2_O signals, respectively.

In contrast, two sets of methylene protons in a 2:1 ratio appear at relatively lower field in DMF-*d*_*7*_, acetone-*d*_*6*_, and THF-*d*_*8*_ at −40 °C. These peaks resolve into three sets at lower temperatures (Figure 3b-d), consistent with an embraced dimeric structure (Scheme 1), in which TMPA spans two copper centers, creating three chemically inequivalent methylene environments. Similarly, the number of aromatic signals increases from three in MeCN (monomer) to eight to 12 in other solvents at −80 °C, indicating reduced symmetry in dimeric species.

Despite the added complexity from substitution, both [(F_2_tmpa)­Cu^I^]­[B­(C_6_F_5_)_4_] and [(MeTFE-tmpa)­Cu^I^]­[B­(C_6_F_5_)_4_] exhibit similar methylene and aromatic splitting patterns and temperature-dependent behavior (Figures S38–S39). Notably, the methyl signal of [(MeTFE-tmpa)­Cu^I^]­[B­(C_6_F_5_)_4_] in THF-*d*_*8*_ resolves into two distinct sets with a 2:1 ratio at low temperature (Figure S40), likely reflecting random distribution of substituted pyridyl arms between bridged or nonbridged positions. This agrees with the X-ray structure of [(F_2_tmpa)_2_Cu^I^_2_]^2+^ which reveals ∼66% fluorine occupancy across the three pyridyl arms, vide infra. Additionally, low-temperature ^19^F NMR spectra in acetone-*d*_*6*_ and THF-*d*_*8*_ (Figures S41–S42) show multiple resonances for the 3-fluoropyridyl and CF_3_ groups, further supporting reduced symmetry and stochastic arm positioning within the embraced dimer structure. Variable-temperature equilibrium analysis revealed that dimer formation is favored by about 2 orders of magnitude upon cooling from room temperature to −80 °C in acetone-*d*_*6*_, corresponding to an approximate standard enthalpy change (Δ*H*°) of −10 kcal·mol^–1^ (Tables S8–S10 and Figures S43–S44).

Interestingly, prior studies reported that TMPA-based Hg­(II) complexes display sharp, distinct proton signals in MeCN-*d*_*3*_ but show severe broadening and unassignable ^1^H NMR spectra in acetone-*d*_*6*_.^5^ While the cause was not identified, the formation of similar embraced multimeric species may account for these observations.

The proposed embraced dimeric structure was further confirmed by single crystal X-ray analysis (Figure 4 and Tables S11–S12), providing definitive evidence of its conformation and intermolecular interactions. Pale yellow crystals of [(tmpa)_2_Cu^I^_2_]­[PF_6_]_2_ and [(F_2_tmpa)_2_Cu^I^_2_]­[B­(C_6_F_5_)_4_]_2_ were grown by slow diffusion of hexanes into THF solutions. Both complexes crystallize in the triclinic space group *P*1̅. In the solid-state, two pairs of TMPA-based chelates define a tetra-coordinate geometry in a flattened tetrahedron around each copper center.

**Figure 4:**
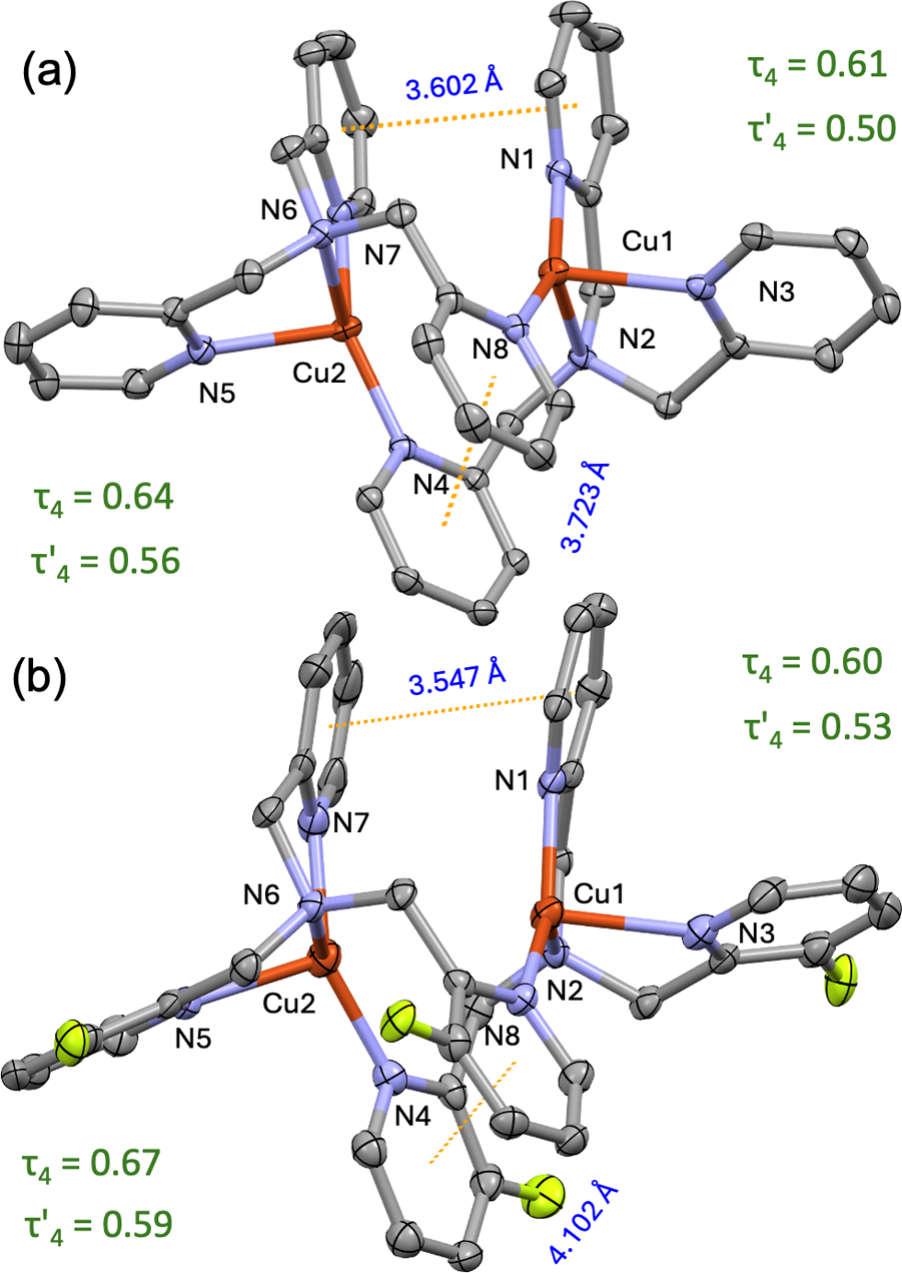
Displacement ellipsoid plot (50% probability level) of the cationic portion of (a) [(tmpa)_2_Cu^I^_2_]­[PF_6_]_2_ and (b) [(F_2_tmpa)_2_Cu^I^_2_]­[B­(C_6_F_5_)_4_]_2_ showing atom labeling. In (b), the two fluorine atoms with 0.66 occupancy across three pyridyl arms are shown arbitrarily.

The τ_4_ ^50^ and τ′_4_ ^56^ values for the two copper centers are provided in Figure 4. Each copper center is ligated by two pyridyl and one tertiary amine nitrogen atoms of a TMPA ligand, while an extended pyridyl arm of the adjacent TMPA occupies the fourth position. This arrangement yields an embraced dimeric structure with an average Cu–N_bridge_ bond length 1.954 Å, which is significantly shorter than the average Cu–N bond length of 2.144 Å, suggesting strong interligand interactions. Both sides of the dimeric cation exhibit high structural similarity (Figures S45–S46), consistent with previous reports.^34^ Additionally, two pairs of π–π interactions are observed: one between two nonbridging arms and another between the two bridging pyridyl rings, positioned at distances of 3.602 Å and 3.723 Å, respectively. These π–π stacking interactions likely contribute to the stabilization of the dimeric species.^57^ A comparable structure was observed for [(F_2_tmpa)_2_Cu^I^_2_]^2+^, with a Cu–N_bridge_ bond length of 1.959 Å and an average Cu–N bond length of 2.129 Å. Strikingly, the statistical refinement of 1H-2F atoms at the 3-positions revealed a fluorine occupancy of 0.66 across the three pyridyl arms, indicating no positional preference for the substituted rings. Despite repeated attempts, we were unable to crystallize [(MeTFE-tmpa)_2_Cu^I^_2_]­[B­(C_6_F_5_)_4_]_2_, likely due to the increased conformational flexibility introduced by the TFE substituent.

In summary, our study underscores the pivotal role of the solvent environment in governing the structural dynamics and speciation of TMPA-based copper­(I) complexes. These solvent-dependent effects critically impact the electron transfer kinetics and thermodynamics, shaping the redox behavior central to mechanistic studies, catalysis, and broader chemical applications.

## Supplementary Material



## Notes

Synthetic and analytical details, including methodologies, electrochemical data, NMR spectra, and crystallographic tables (PDF)

## #

Department of Chemistry, Virginia Tech, Blacksburg, Virginia 24061, United States
