# Supplementary material for: Dimerization in TMPA-Based Copper(I) Complexes: Implications for Redox Kinetics and Thermodynamics
Source: Inorg Chem. 2025 Jun 16;64(25):12416–22. doi: 10.1021/acs.inorgchem.5c01099 (PMC12216234; doi:10.1021/acs.inorgchem.5c01099)
Supplement: Supplementary file 1 [file ic5c01099_si_001.pdf]

Supporting Information

Dimerization in TMPA-Based Copper(I) Complexes: Implications for Redox Kinetics and Thermodynamics

Marcos Tapia, Shyam K. Pahari, Sandip Das, Firoz S. T. Khan, and Shabnam Hematian*

Department of Chemistry and Biochemistry, University of North Carolina at Greensboro, Greensboro, NC 27402, USA

* Correspondence: s_hemati@uncg.edu; Tel.: +1-336-334-5867

Table of Contents

Experimental Section S2

1. General methods S2
2. Electrochemistry S3
   a. Uncompensated resistance (R_u) and impedance measurements S3
   b. Cyclic voltammetry of Cu(I) complexes S3
   c. Estimation and validation of dimer-monomer concentrations S4
   d. Diffusion coefficient measurement S4
   e. Estimation of heterogeneous electron transfer rate constants S5
   f. Electrochemical properties of **[(tmpa)Cu^I]$^+$**, **[(F$_2$tmpa)Cu^I]$^+$**, and **[(MeTFE-tmpa)Cu^I]$^+$** as a function of solvent properties S6
   g. Kinetic modeling and determination of the dissociation rate constant for the oxidized dimer of **[(tmpa)Cu^I]$^+$** in acetone. S15
   h. Effect of electrolyte properties on monomer-dimer equilibria ..... S**Error! Bookmark not defined.**
   i. Digital simulation of cyclic voltammograms S18
3. Variable-temperature NMR in varying solvents S21
   a. Thermodynamic analysis of dimerization from VT-NMR data S24
4. Single crystal X-ray diffraction S26

References S28

Experimental Section

1. General methods

All commercially available chemicals were of the highest purity and used without further purification unless otherwise specified. Acetonitrile (MeCN), dichloromethane (DCM), tetrahydrofuran (THF), 2-methyltetrahydrofuran (MeTHF), propylene carbonate (PC), acetone, and dimethylformamide (DMF) were obtained from Sigma-Aldrich. Methanol (CH_3OH) and diethyl ether were obtained from Fisher Chemicals. Potassium *tetrakis*(pentafluorophenyl)borate, $K[B(C_6F_5)_4]$, (99.9%) was purchased from Boulder Scientific Company. *Tetra-n*-butylammonium hexafluorophosphate (98%) was obtained from Oakwood Chemicals and recrystallized twice from ethanol/water. Deuterated solvents ($CDCl_3$, MeCN-*d3*, DMF-*d7*, acetone-*d6*, and THF-*d8*) were acquired from Cambridge Isotope Laboratories. Commercial ACS-grade solvents were used for chromatography and extractions. All solvents were purified using an Innovative Technologies or Inert PureSolv Micro solvent purification system before being used in reactions and characterizations. Following purification, solvents were deoxygenated by bubbling Argon for 1 hour and subsequently stored over 3 Å or 5 Å molecular sieves for a minimum of 72 hours inside the glovebox before use. Air and moisture-sensitive compounds were manipulated under a nitrogen (N_2) atmosphere in an OMNI-Lab inert atmosphere glovebox (O_2 < 0.5 ppm and H_2O < 0.5 ppm) or within a dry, oxygen-free argon environment using standard Schlenk-line techniques. All deuterated solvents, sealed in ampules, were brought into the glovebox without opening. Inside the glovebox, the solvents were transferred to vials and then bubbled at least 100 times using a 2 mL glass pipette to equilibrate with the glovebox atmosphere. The compounds **$[(tmpa)Cu^I][B(C_6F_5)_4]$**,[1] **$[(F_2tmpa)Cu^I][B(C_6F_5)_4]$**,[1] **$[(MeTFE\text{-}tmpa)Cu^I][B(C_6F_5)_4]$**,[1] $[(tmpa)Cu^{II}(Cl)][B(C_6F_5)_4]$,[2] $[Ag^I(MeCN)_4][B(C_6F_5)_4]$,[3] and *tetra-n*-butylammonium *tetrakis*(pentafluorophenyl)borate ($[(n\text{-}Bu)_4N][B(C_6F_5)_4]$)[3] were synthesized and characterized following methods previously reported by our laboratory.

Variable-temperature ^1H and ^{19}F nuclear magnetic resonance (NMR) measurements were performed on a Jeol 400 MHz spectrometer equipped with a Jeol NM-51740DW10 cryostat, which utilizes liquid nitrogen for cooling to the desired temperature. The chemical shifts for ^1H NMR were referenced against tetramethyl-silane (TMS, δ = 0.00 ppm), while ^{19}F chemical shifts were referenced against the *p*-fluorines of $[B(C_6F_5)_4]^-$ (*p*-BArF) at -164.28 ppm.

a. Synthesis of **$[(tmpa)Cu^{II}][B(C_6F_5)_4]_2$**

Inside the glove box, a solution of $[Ag^I(MeCN)_4][B(C_6F_5)_4]$ (130.0 mg, 0.13 mmol) in THF (2 ml) was added dropwise to a solution of $[(tmpa)Cu^{II}(Cl)][B(C_6F_5)_4]$ (106.0 mg, 0.108 mmol) in THF (2 ml) in a 100 ml Schlenk flask. Upon addition, a white precipitate of AgCl immediately formed, and the solution color changed from torque to aqua blue. The flask was sealed with a septum and removed from the glove box under a nitrogen atmosphere, then sonicated for 2.5 h to ensure complete removal of chloride as AgCl. The reaction mixture was returned to the glove box and filtered through Celite to yield a clear teal blue solution of **$[(tmpa)Cu^{II}][B(C_6F_5)_4]_2$**. THF was removed under vacuum to afford a semi-crystalline teal blue solid. Due to the use of excess $[Ag^I(MeCN)_4][B(C_6F_5)_4]$ for chloride abstraction, minor $[Ag^I(MeCN)_4][B(C_6F_5)_4]$ and $[(tmpa)Cu^{II}(Cl)][B(C_6F_5)_4]$ impurities may remain. The estimated purity is 80-85%, according to the chloro-to-copper charge transfer band at 300 nm and electrochemical measurements. Crude Yield: 144 mg (86%). UV-vis in THF [λ_{max}, nm (ε_{max}, $M^{-1}cm^{-1}$)]: 310 (937), 692 (61), 877 (205). The ε values were corrected for estimated impurity.

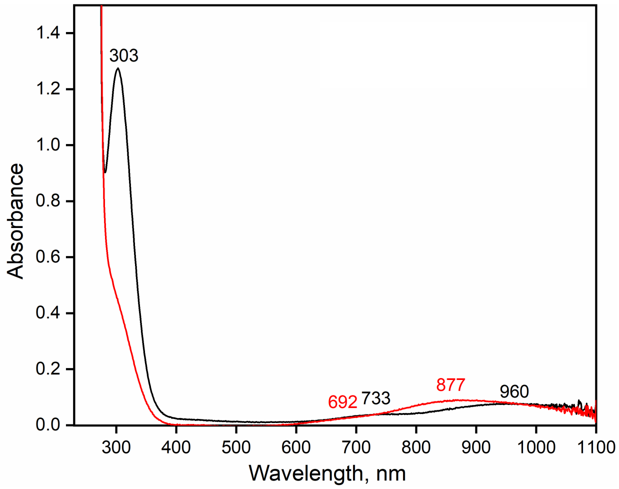

Figure S1. Room-temperature UV-vis spectra of 0.4 mM THF solutions of **[(tmpa)Cu^{II}][B(C_6F_5)$_4$]$_2$** (red) and [(tmpa)Cu^{II}(Cl)][B(C_6F_5)$_4$] (black) recorded in a 1-cm pathlength cuvette.

2. Electrochemistry

a. Uncompensated resistance (R_u) and impedance measurements

Prior to each cyclic voltammetry (CV) experiment, the uncompensated resistance was measured using potentio electrochemical impedance spectroscopy (PEIS) to correct for internal resistance drop of voltage in the electrolyte solution. A three-electrode setup was employed using the same electrolyte/solvent combinations as were used in corresponding CV experiments. The frequency was scanned from 1 MHz to 100 mHz at solution open circuit potential and the real impedance value observed at 1 MHz was considered the uncompensated resistance value for that solution. To avoid instabilities in the potentiostat, the *i*R drop was corrected for only 85% of the uncompensated solution resistance (R_u) during the cyclic voltammetry measurements through positive feedback using the Bio-Logic EC-Lab software. The R_u values for each solvent are listed below in Table S1.

Table S1. Uncompensated resistance (R_u) values measured in each solvent using 100 mM [(*n*-Bu)$_4$N][B(C_6F_5)$_4$] as the supporting electrolyte.

Solvents	R_u value (Ω)
PC	61
MeCN	50
DMF	182-228
MeTHF	250
Acetone	85-165
THF	200-326

b. Cyclic voltammetry of Cu(I) complexes

All CV experiments were performed at room temperature in a N_2 filled glovebox (< 0.5 ppm O_2 and 0.5 ppm H_2O) on a Biologic SP-200 potentiostat using a custom-made 3-electrode cell comprised of 3.0 mm diameter glassy carbon working electrode, a leak-free Ag/AgCl reference electrode (Innovative Instrument inc.), and a 2-mm diameter carbon rod counter electrode. The Ag/AgCl reference electrodes were stored in a saturated KCl solution. Before performing CV experiments in organic solutions, the impedance of the Ag/AgCl reference electrodes was measured in a 100 mM aqueous KCl solution using impedance spectroscopy in a 2-electrode setup. The frequency was scanned from 200 kHz to 1 Hz, and the real impedance value observed at 200 kHz was considered the impedance for that particular Ag/AgCl electrode. An impedance under 15,000 Ohm was considered suitable for use. Prior to each experiment, the glassy carbon electrode was polished with 0.05 μm alumina slurry in ultra-pure water, sonicated in water briefly, dried with acetone followed by dry N_2, and transferred to the glovebox. All the electrodes were once again washed with dry solvent inside the glovebox to eliminate any potential moisture adsorbed on the surface.

Each sample prepared for CV contained 2 mM of Cu(I) center. Samples were prepared in corresponding solvents with 100 mM $[(n\text{-Bu})_4N][B(C_6F_5)_4]$ as supporting electrolytes 30 min prior to data collection. The reduction potentials were reported against Ag/AgCl reference electrode and validated by measuring the $E_{½}$ of ferrocene, used as an internal standard. For variable scan rate experiments, scan rates were varied at 1000, 500, 200, 100, 50, 25, and 10 mV s^{-1}, beginning with positive scan direction from open circuit potentials which range between -0.28 V to 0.3 V vs. Ag/AgCl.

c. Estimation and validation of dimer-monomer concentrations

CV experiments were performed using solutions with known total concentration of Cu(I) centers, but the monomer-dimer distribution could not be directly measured. To estimate the relative populations, the charge passed during oxidation of each species was calculated by integrating current-time segments from the CV trace, accounting for the two-electron transfer of the dimer with two copper centers vs. the one-electron transfer of the monomer with one copper center. To validate this method, a 2 mM solution of **$[(tmpa)Cu^I][B(C_6F_5)_4]$** with 100 mM $[(n\text{-Bu})_4N][B(C_6F_5)_4]$ in acetone was analyzed by both CV and chronoamperometry. In chronoamperometry, step potentials were applied to oxidize the dimer alone or both species, and the corresponding charge was determined by integrating the current decay, correcting for double-layer charging. As shown in Figure S2, both CV and chronoamperometric integration yielded a consistent monomer-to-dimer charge ratio of ~3:1, supporting the reliability of the CV-based speciation analysis.

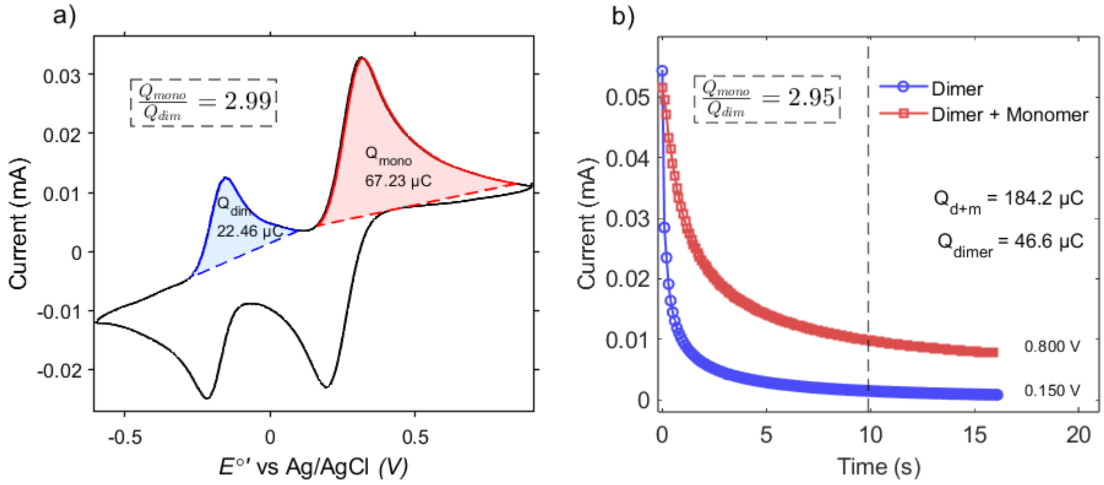

Figure S2. Quantification of monomer and dimer by CV and validation by chronoamperometry. (a) Cyclic voltammogram collected at 100 mV s^{-1} for a 2 mM solution of **$[(tmpa)Cu^I][B(C_6F_5)_4]$** in acetone with 100 mM $[(n\text{-}Bu)_4N][B(C_6F_5)_4]$ supporting electrolyte. The one-electron monomer and two-electron dimer oxidation waves are shaded with red and blue, respectively. Regions under each peak indicate the integrated areas used to calculate the monomer-dimer ratio. (b) Current-time decay corresponding to potentials held at 0.150 V vs. Ag/AgCl (blue trace) chosen to oxidize only the dimer and at 0.800 V vs. Ag/AgCl (red trace) chosen to oxidize both dimer and monomer. The solution was mixed well between each experiment. The resulting charge ratio from chronoamperometry agrees with the ~3:1 monomer:dimer ratio obtained from CV.

d. Diffusion coefficient measurement

The diffusion coefficient (D_0) for each monomer and dimer species was calculated using Randles-Sevcik equation from variable scan rate CVs. EC-Lab software was used to determine the anodic and cathodic peak currents, which were then plotted against the square root of scan rate.

The peak current is related to the diffusion coefficient according to the Randles-Sevcik equation,

$$i_p = 0.4463nFAC^0\left(\frac{nF\upsilon D}{RT}\right)^{1/2} = \left[0.4463nFAC^0\left(\frac{nFD}{RT}\right)^{1/2}\right]\upsilon^{1/2} \qquad \text{……………………} \quad \text{(Eq. 1)}$$

Where i_p is peak current (A), F is Faraday's constant (96485 C mol^{-1}), R is the ideal gas constant (J mol^{-1} K^{-1}), T is temperature (K), n is the number of electrons in the redox reaction, C_0 is concentration (mol cm^{-3}), A is the surface area of the electrode (cm^2), υ is the scan rate (V s^{-1}), and D_0 is the diffusion coefficient (cm^2 s^{-1}). Rearranging the equation,

$$slope = \left[0.4463nFAC^0\left(\frac{nFD}{RT}\right)^{\frac{1}{2}}\right] \quad \ldots\ldots\ldots\ldots\ldots\ldots\ldots \quad \text{(Eq. 2)}$$

From the slope, D_0 can be calculated as,

$$D_0 = \left(\frac{slope}{0.4463nFAC^0}\right)^2 \frac{RT}{nF} \quad \ldots\ldots\ldots\ldots\ldots\ldots\ldots \quad \text{(Eq. 3)}$$

Diffusion coefficient values are listed in Table S3. While diffusion coefficients decrease monotonically with increasing solvent viscosity, the relative ordering of D_0 values for monomeric and dimeric species varies by solvent. This behavior reflects a nontrivial interplay between hydrodynamic drag and solvation effects. In strongly coordinating solvents, the monomeric complex, due to solvent binding and exchange, may possess a larger effective radius than the more compact, solvent-shielded dimer. These solvation differences are nuanced and context-dependent, accounting for the solvent- and complex-specific diffusion behavior observed (Figure S31).

e. Estimation of heterogeneous electron transfer rate constants

The standard rate constant, k^0, was estimated using the Nicolson method.[4] The relationship between the k^0 and the Nicolson dimensionless number (ψ) is given by the Eq.

$$k^0 = \left(\frac{\pi D_0 nFv}{RT}\right)^{\frac{1}{2}}.\psi \quad \ldots\ldots\ldots\ldots\ldots\ldots\ldots \quad \text{(Eq. 4)}$$

Symbols have their usual meaning as defined for equation 1. The above equation can be rearranged in the form of a linear equation as follows:

$$\psi = k^0\left(\frac{\pi D_0 nF}{RT}\right)^{-\frac{1}{2}}.v^{-\frac{1}{2}} \quad \ldots\ldots\ldots\ldots\ldots\ldots\ldots \quad \text{(Eq. 5)}$$

Figure 3 from Nicolson's classic paper[4] provides a method to obtain the dimensionless parameter ψ for each CV curve at various scan rates, utilizing the peak potential separation (ΔE_p).

Finally, the Nicholson dimensionless parameter corresponding to peak separation for each scan rate was plotted against the inverse square root of the scan rate in V s^{-1}. We determined k^0 only for datasets whose peak-potential separations (ΔE_p) fall within Nicholson method's applicable range and exhibit a measurable scan-rate dependence. Data outside this window were excluded, and the corresponding entries in Table S4 are left blank.

The resulting slope was used to estimate the k^0 using the following equation.

$$k^0 = slope \times \left(\frac{\pi D_0 nF}{RT}\right)^{\frac{1}{2}} \quad \ldots\ldots\ldots\ldots\ldots\ldots\ldots \quad \text{(Eq. 6)}$$

f. Electrochemical properties of ***[(tmpa)Cu^{I}]$^+$***, ***[(F$_2$tmpa)Cu^{I}]$^+$***, *and* ***[(MeTFE-tmpa)Cu^{I}]$^+$*** *as a function of solvent properties*

Table S2. Reduction potential ($E_{½}$), peak separation (ΔE_p), and anodic to cathodic peak ratio (i_{pa}/i_{pc}) of monomer and dimer species of each complex in various solvents measured at scan rate of 100 mV s^{-1}. The $E_{½}$ values are reported in V *vs.* Ag/AgCl and ΔE_p values are reported in mV.

Solvent	[(F$_2$tmpa)Cu^I]$^+$						[(tmpa)Cu^I]$^+$						[(MeTFE-tmpa)Cu^I]$^+$					
	Dimer			Monomer			Dimer			Monomer			Dimer			Monomer		
	$E_{½}$	ΔE_p	i_{pa}/i_{pc}	$E_{½}$	ΔE_p	i_{pa}/i_{pc}	$E_{½}$	ΔE_p	i_{pa}/i_{pc}	$E_{½}$	ΔE_p	i_{pa}/i_{pc}	$E_{½}$	ΔE_p	i_{pa}/i_{pc}	$E_{½}$	ΔE_p	i_{pa}/i_{pc}
PC	-0.26	83	1.15	0.17	174	1.10	-0.34	70	1.09	0.09	137	1.03	-0.34	80	1.05	0.04	151	1.06
MeCN	-0.21	39	1.15	0.16	79	0.99	-0.29	36	0.99	0.05	74	0.99	-0.32	41	1.09	-0.05	79	1.03
DMF	-0.22	49	1.33	-0.03	105	0.90	-0.30	42	1.2	-0.11	86	0.92	-0.34	43	1.12	-0.14	89	0.90
MeTHF	-0.13	96	0.86	0.35	304	1.20	-0.23	93	0.54	0.33	289	1.65	-0.27	92	0.82	0.24	257	1.14
Acetone	-0.22	69	0.99	0.21	136	0.86	-0.30	68	1.01	0.14	124	0.96	-0.30	74	0.80	0.09	169	1.02
THF	-0.14	97	0.92	0.41	196	1.04	-0.25	85	0.93	0.36	135	1.34	-0.32	72	0.97	0.29	132	1.11

Table S3. Diffusion coefficient values (D_0) of monomer and dimer species of each complex across various solvents.

Solvent	Diffusion coefficient (D_0) × 10^6 (cm^2.s^{-1})											
	[(F$_2$tmpa)Cu^I]$^+$				[(tmpa)Cu^I]$^+$				[(MeTFE-tmpa)Cu^I]$^+$			
	Dimer		Monomer		Dimer		Monomer		Dimer		Monomer	
	Anodic	Cathodic	Anodic	Cathodic	Anodic	Cathodic	Anodic	Cathodic	Anodic	Cathodic	Anodic	Cathodic
PC	1.80	1.17	0.39	0.30	0.64	0.61	0.35	0.29	2.53	1.93	0.63	0.44
MeCN	13.48	11.41	6.78	6.64	8.89	8.74	6.85	6.78	7.00	8.12	6.48	6.09
DMF	1.35	1.06	1.66	1.93	4.19	3.85	1.94	2.20	1.96	1.14	1.27	1.26
MeTHF	0.610	0.926	1.30	0.941	0.328	1.87	1.85	0.316	0.383	0.962	1.77	0.916
Acetone	1.41	1.42	2.71	2.69	8.79	8.48	1.13	1.09	0.87	1.1	1.28	1.21
THF	0.577	0.669	1.49	1.11	0.477	0.680	2.14	1.50	0.814	0.856	1.58	1.24

Table S4. Electron transfer rate constant (k^0) values measured using Nicholson method.

Solvent	Electron transfer rate constants k^0 × 10^3 (cm.s^{-1})					
	[(F$_2$tmpa)Cu^I]$^+$		[(tmpa)Cu^I]$^+$		[(MeTFE-tmpa)Cu^I]$^+$	
	Dimer	Monomer	Dimer	Monomer	Dimer	Monomer
PC	–	0.332	–	0.496	–	0.762
MeCN	–	9.904	–	27.98	–	20.33
DMF	–	3.021	–	5.407	–	2.626
MeTHF	4.221	–	14.08	0.406	2.220	0.355
Acetone	14.99	1.92	34.05	1.728	13.27	0.946
THF	3.904	1.048	4.227	1.876	9.969	1.254

k^0 values are not reported for the species with the corresponding ψ beyond useful range for Nicholson method.

Table S5. Relevant solvent parameters.

Solvents	Donor number (DN)	Acceptor number (AN)	Dielectric constant (ε)	Dipole moment (μ), D
PC	15.1 [a]	18.3 [a]	65.5 [b]	5.36 [b]
MeCN	14.1 [c]	18.9 [c]	36 [c]	3.44 [c]
DMF	26.6 [c]	16 [c]	37.5 [c]	3.86 [c]
MeTHF	18 [d]	8 [d]	6.97 [d]	1.38 [d]
Acetone	17 [a]	12.5 [a]	20.7 [a]	2.88 [a]
THF	20 [c]	8 [c]	7.4 [c]	1.7 [c]

[a] From ref. 5, [b] from ref. 6, [c] From ref. 7, [d] from ref. 8. The AN is not reported for MeTHF, although it can be inferred to be close to values reported for tetrahydrofuran (8.0) and diethyl ether (3.9).

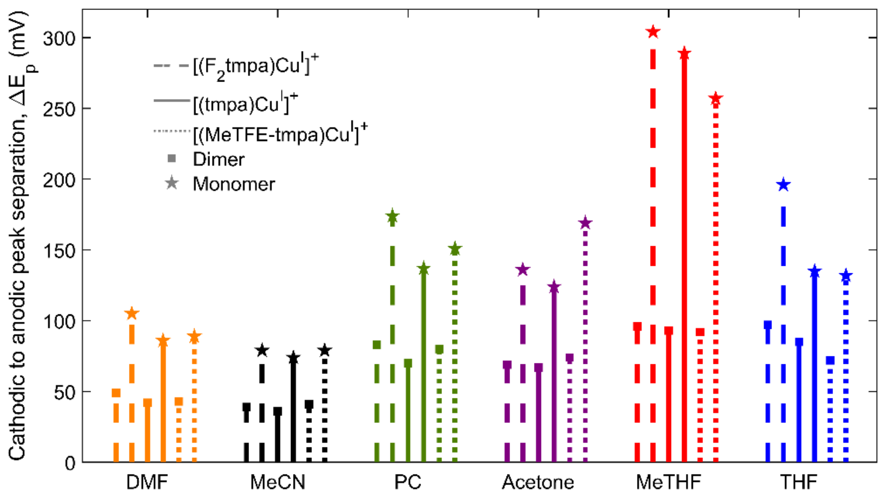

Figure S3. Comparison of cathodic to anodic peak separation for monomer and dimer species across various solvents. Peak separation values were obtained from cyclic voltammetry at a scan rate of 100 mV s^{-1}. The dimer species exhibit a two-electron redox process, resulting in a peak-to-peak separation that is approximately half of that observed for the monomer, which undergo one-electron redox process.

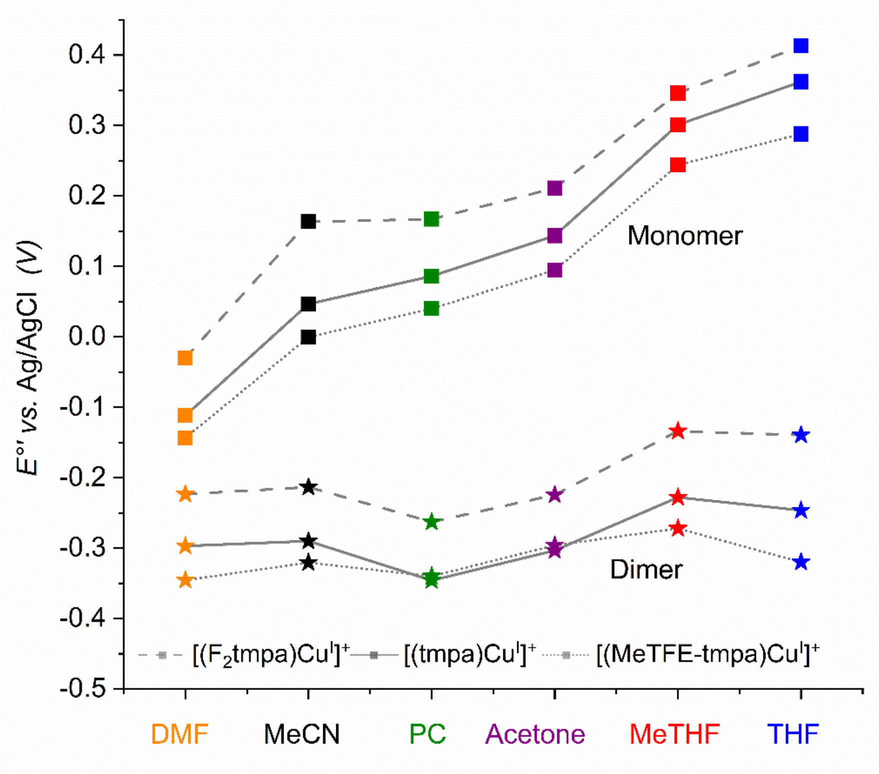

Figure S4. Plot of reduction potential ($E_{½}$) of monomer and dimer species for all three complexes in various solvents.

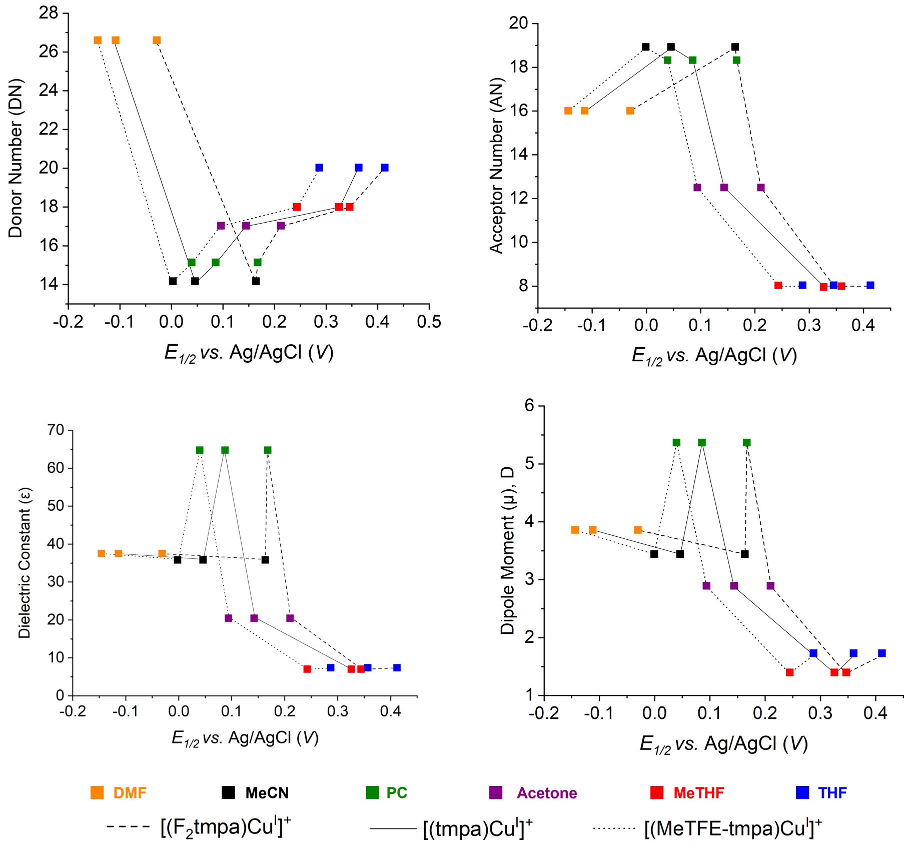

Figure S5. Correlations between the reduction potential of monomer species of complexes with various solvent parameters.

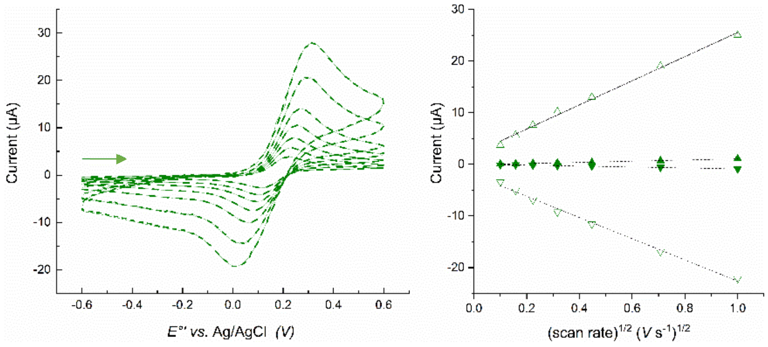

Figure S6. (Left) Cyclic voltammogram of 2 mM **[(F$_2$tmpa)Cu^I]$^+$** in PC with 100 mM [(*n*-Bu)$_4$N][B(C$_6$F$_5$)$_4$] as the supporting electrolyte at variable scan rates. Working electrode: 3-mm glassy carbon, reference electrode: Ag/AgCl, counter electrode: carbon rod. (Right) Corresponding Randles-Sevcik plot.

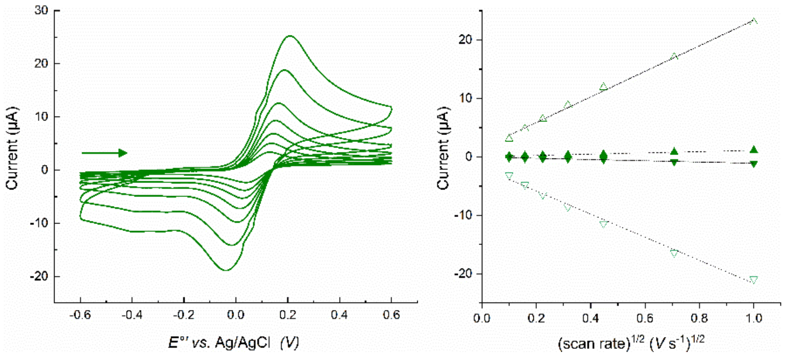

Figure S7. (Left) Cyclic voltammogram of 2 mM **[(tmpa)Cu^{I}]$^+$** in PC with 100 mM [(*n*-Bu)$_4$N][B(C_6F_5)$_4$] as the supporting electrolyte at variable scan rates. Working electrode: 3-mm glassy carbon, reference electrode: Ag/AgCl, counter electrode: carbon rod. (Right) Corresponding Randles-Sevcik plot.

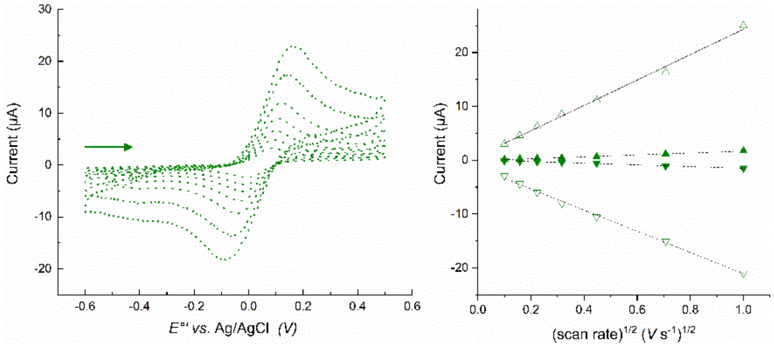

Figure S8. (Left) Cyclic voltammogram of 2 mM **[(MeTFE-tmpa)Cu^{I}]$^+$** in PC with 100 mM [(*n*-Bu)$_4$N][B(C_6F_5)$_4$] as the supporting electrolyte at variable scan rates. Working electrode: 3-mm glassy carbon, reference electrode: Ag/AgCl, counter electrode: carbon rod. (Right) Corresponding Randles-Sevcik plot.

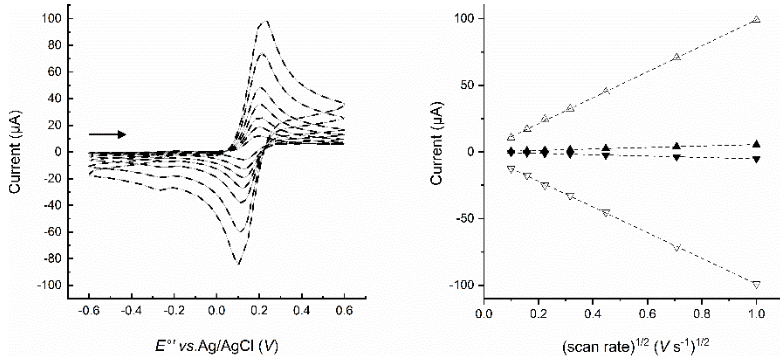

Figure S9. (Left) Cyclic voltammogram of 2 mM **[(F$_2$tmpa)Cu^{I}]$^+$** in MeCN with 100 mM [(*n*-Bu)$_4$N][B(C_6F_5)$_4$] as the supporting electrolyte at variable scan rates. Working electrode: 3-mm glassy carbon, reference electrode: Ag/AgCl, counter electrode: carbon rod. (Right) Corresponding Randles-Sevcik plot.

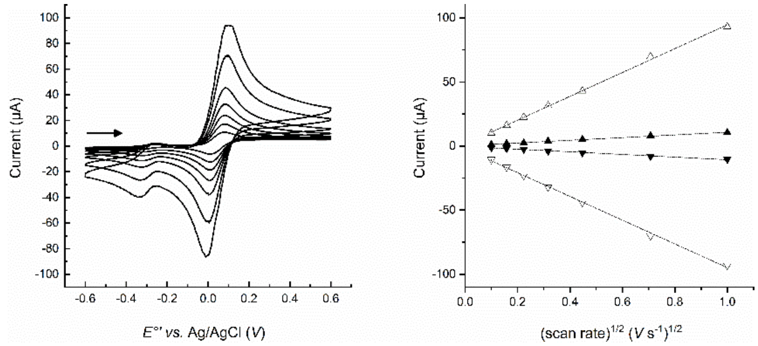

Figure S10. (Left) Cyclic voltammogram of 2 mM **[(tmpa)Cu^{I}]$^+$** in MeCN with 100 mM [(*n*-Bu)$_4$N][B(C_6F_5)$_4$] as the supporting electrolyte at variable scan rates. Working electrode: 3-mm glassy carbon, reference electrode: Ag/AgCl, counter electrode: carbon rod. (Right) Corresponding Randles-Sevcik plot.

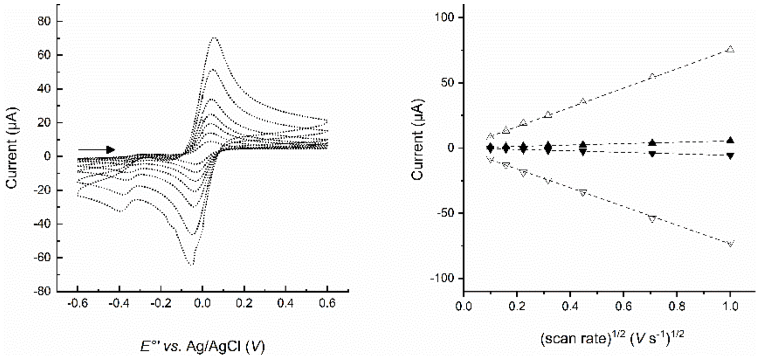

Figure S11. (Left) Cyclic voltammogram of 2 mM **[(MeTFE-tmpa)Cu^{I}]$^{+}$** in MeCN with 100 mM [(*n*-Bu)$_4$N][B(C_6F_5)$_4$] as the supporting electrolyte at variable scan rates. Working electrode: 3-mm glassy carbon, reference electrode: Ag/AgCl, counter electrode: carbon rod. (Right) Corresponding Randles-Sevcik plot.

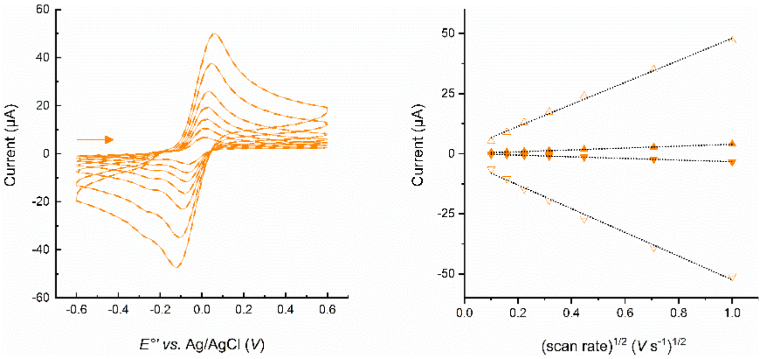

Figure S12. (Left) Cyclic voltammogram of 2 mM **[(F$_2$tmpa)Cu^{I}]$^{+}$** in DMF with 100 mM [(*n*-Bu)$_4$N][B(C_6F_5)$_4$] as the supporting electrolyte at variable scan rates. Working electrode: 3-mm glassy carbon, reference electrode: Ag/AgCl, counter electrode: carbon rod. (Right) Corresponding Randles-Sevcik plot.

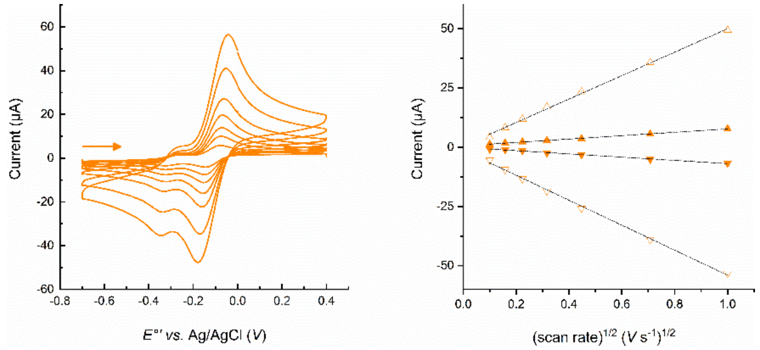

Figure S13. (Left) Cyclic voltammogram of 2 mM **[(tmpa)Cu^{I}]$^{+}$** in DMF with 100 mM [(*n*-Bu)$_4$N][B(C_6F_5)$_4$] as the supporting electrolyte at variable scan rates. Working electrode: 3-mm glassy carbon, reference electrode: Ag/AgCl, counter electrode: carbon rod. (Right) Corresponding Randles-Sevcik plot.

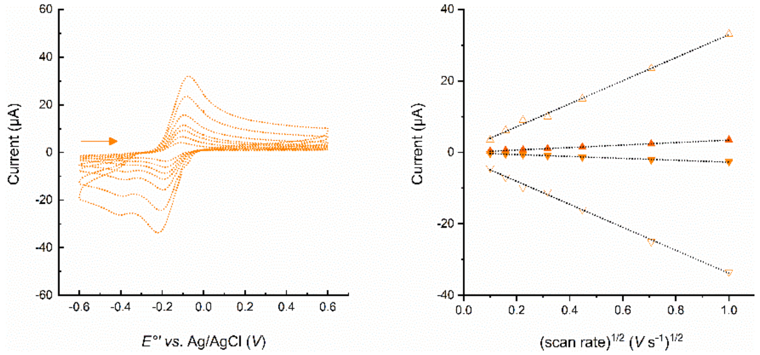

Figure S14. (Left) Cyclic voltammogram of 2 mM **[(MeTFE-tmpa)Cu^{I}]$^{+}$** in DMF with 100 mM [(*n*-Bu)$_4$N][B(C_6F_5)$_4$] as the supporting electrolyte at variable scan rates. Working electrode: 3-mm glassy carbon, reference electrode: Ag/AgCl, counter electrode: carbon rod. (Right) Corresponding Randles-Sevcik plot.

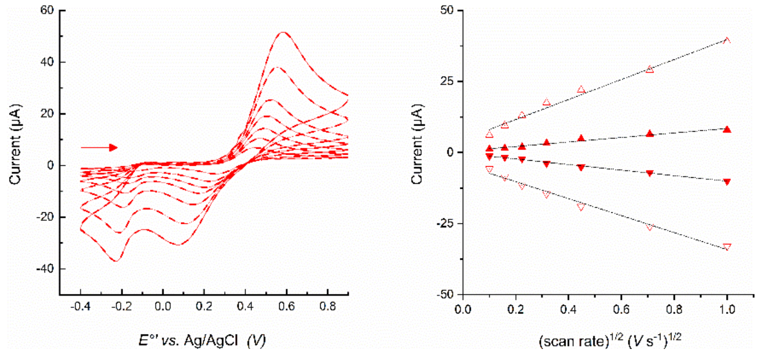

Figure S15. (Left) Cyclic voltammogram of 2 mM **[(F$_2$tmpa)Cu^I]$^+$** in MeTHF with 100 mM [(*n*-Bu)$_4$N][B(C$_6$F$_5$)$_4$] as the supporting electrolyte at variable scan rates. Working electrode: 3-mm glassy carbon, reference electrode: Ag/AgCl, counter electrode: carbon rod. (Right) Corresponding Randles-Sevcik plot.

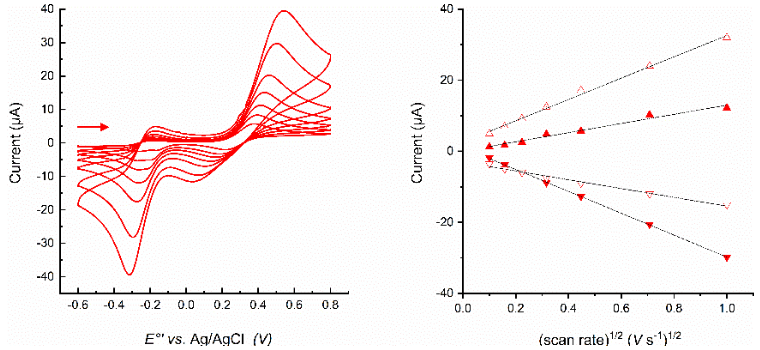

Figure S16. (Left) Cyclic voltammogram of 2 mM **[(tmpa)Cu^I]$^+$** in MeTHF with 100 mM [(*n*-Bu)$_4$N][B(C$_6$F$_5$)$_4$] as the supporting electrolyte at variable scan rates. Working electrode: 3-mm glassy carbon, reference electrode: Ag/AgCl, counter electrode: carbon rod. (Right) Corresponding Randles-Sevcik plot.

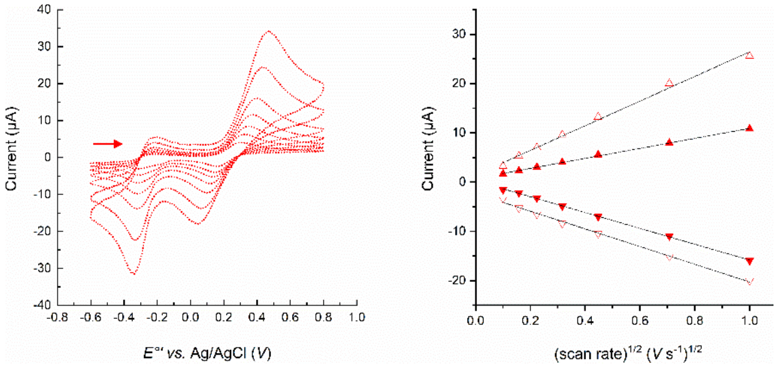

Figure S17. (Left) Cyclic voltammogram of 2 mM **[(MeTFE-tmpa)Cu^I]$^+$** in MeTHF with 100 mM [(*n*-Bu)$_4$N][B(C$_6$F$_5$)$_4$] as the supporting electrolyte at variable scan rates. Working electrode: 3-mm glassy carbon, reference electrode: Ag/AgCl, counter electrode: carbon rod. (Right) Corresponding Randles-Sevcik plot.

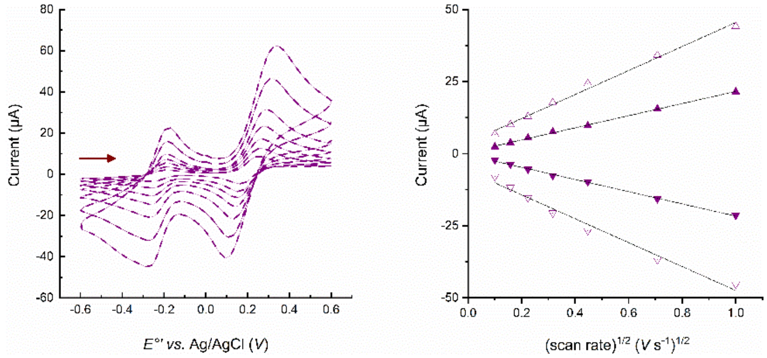

Figure S18. (Left) Cyclic voltammogram of 2 mM **[(F$_2$tmpa)Cu^I]$^+$** in acetone with 100 mM [(*n*-Bu)$_4$N][B(C$_6$F$_5$)$_4$] as the supporting electrolyte at variable scan rates. Working electrode: 3-mm glassy carbon, reference electrode: Ag/AgCl, counter electrode: carbon rod. (Right) Corresponding Randles-Sevcik plot.

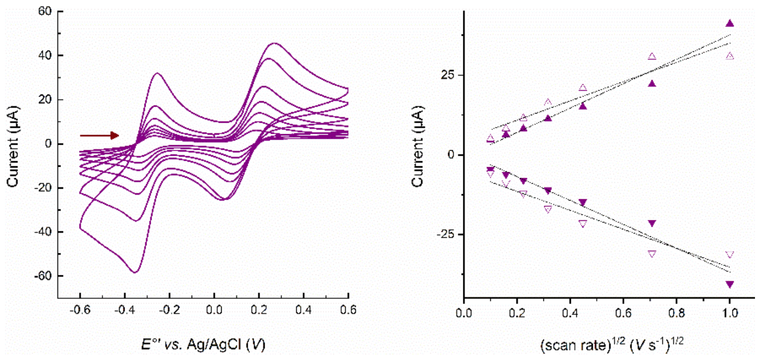

Figure S19. (Left) Cyclic voltammogram of 2 mM **[(tmpa)Cu^{I}]$^{+}$** in acetone with 100 mM [(*n*-Bu)$_4$N][B(C$_6$F$_5$)$_4$] as the supporting electrolyte at variable scan rates. Working electrode: 3-mm glassy carbon, reference electrode: Ag/AgCl, counter electrode: carbon rod. (Right) Corresponding Randles-Sevcik plot.

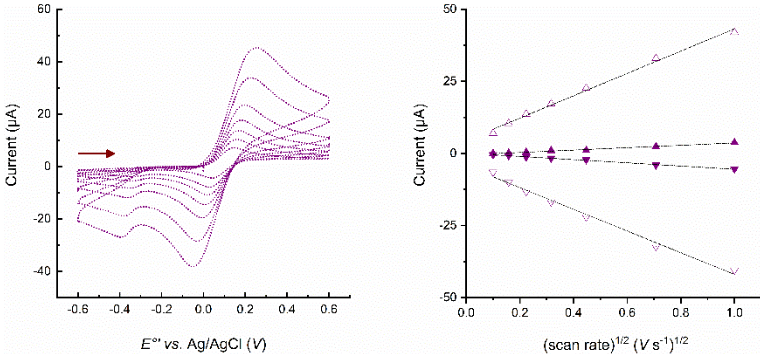

Figure S20. (Left) Cyclic voltammogram of 2 mM **[(MeTFE-tmpa)Cu^{I}]$^{+}$** in acetone with 100 mM [(*n*-Bu)$_4$N][B(C$_6$F$_5$)$_4$] as the supporting electrolyte at variable scan rates. Working electrode: 3-mm glassy carbon, reference electrode: Ag/AgCl, counter electrode: carbon rod. (Right) Corresponding Randles-Sevcik plot.

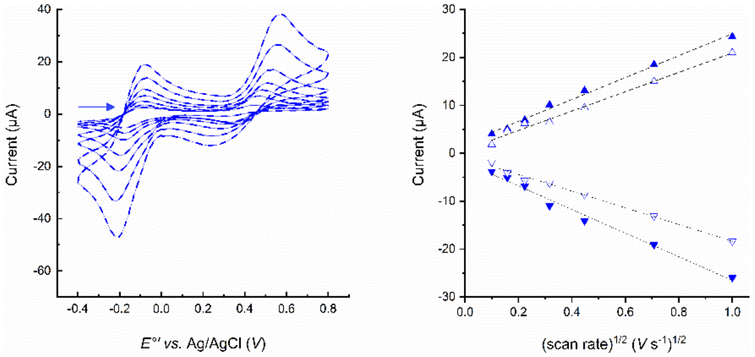

Figure S21. (Left) Cyclic voltammogram of 2 mM **[(F$_2$tmpa)Cu^{I}]$^{+}$** in THF with 100 mM [(*n*-Bu)$_4$N][B(C$_6$F$_5$)$_4$] as the supporting electrolyte at variable scan rates. Working electrode: 3-mm glassy carbon, reference electrode: Ag/AgCl, counter electrode: carbon rod. (Right) Corresponding Randles-Sevcik plot.

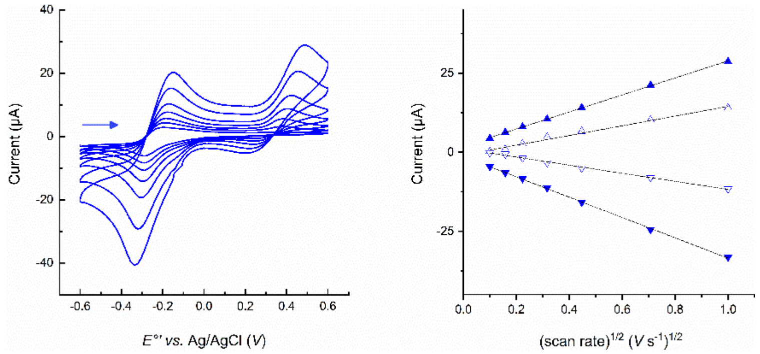

Figure S22. (Left) Cyclic voltammogram of 2 mM **[(tmpa)Cu^{I}]$^{+}$** in THF with 100 mM [(*n*-Bu)$_4$N][B(C$_6$F$_5$)$_4$] as the supporting electrolyte at variable scan rates. Working electrode: 3-mm glassy carbon, reference electrode: Ag/AgCl, counter electrode: carbon rod. (Right) Corresponding Randles-Sevcik plot.

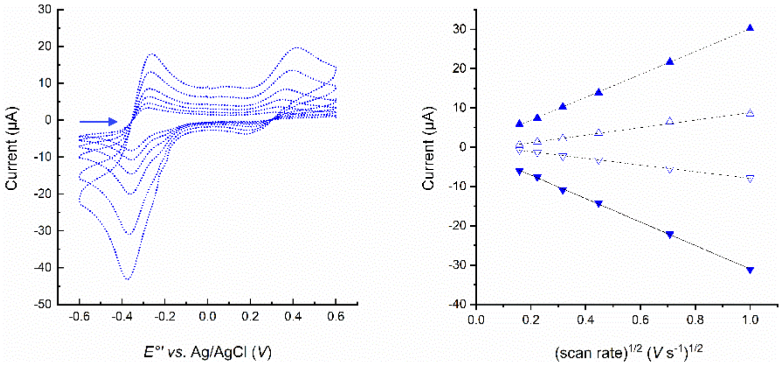

Figure S23. (Left) Cyclic voltammogram of 2 mM **[(MeTFE-tmpa)Cu^I]$^+$** in THF with 100 mM [(n-Bu)$_4$N][B(C_6F_5)$_4$] as the supporting electrolyte at variable scan rates. Working electrode: 3-mm glassy carbon, reference electrode: Ag/AgCl, counter electrode: carbon rod. (Right) Corresponding Randles-Sevcik plot.

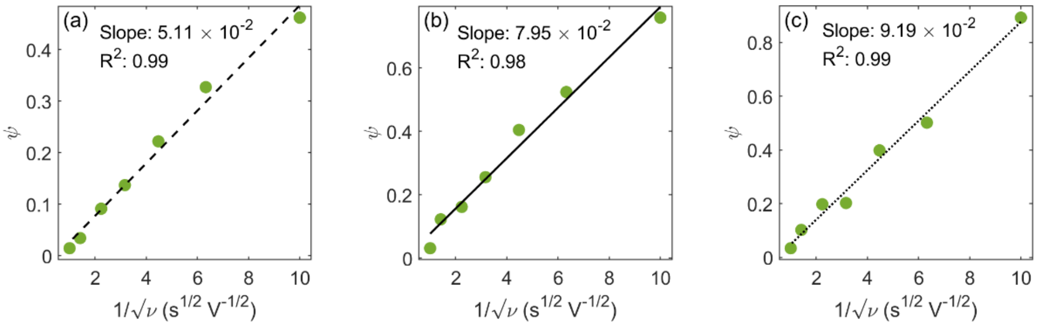

Figure S24. Plot of Nicholson dimensionless parameter (ψ) versus (ν)$^{-1/2}$ for monomeric species of a) **[(F$_2$tmpa)Cu^I]$^+$**, b) **[(tmpa)Cu^I]$^+$**, and c) **[(MeTFE-tmpa)Cu^I]$^+$** in PC.

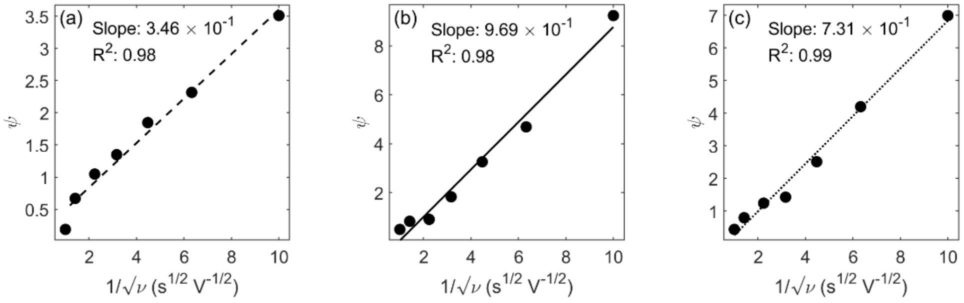

Figure S25. Plot of Nicholson dimensionless parameter (ψ) versus (ν)$^{-1/2}$ for monomeric species of a) **[(F$_2$tmpa)Cu^I]$^+$** b) **[(tmpa)Cu^I]$^+$** and c) **[(MeTFE-tmpa)Cu^I]$^+$** in MeCN.

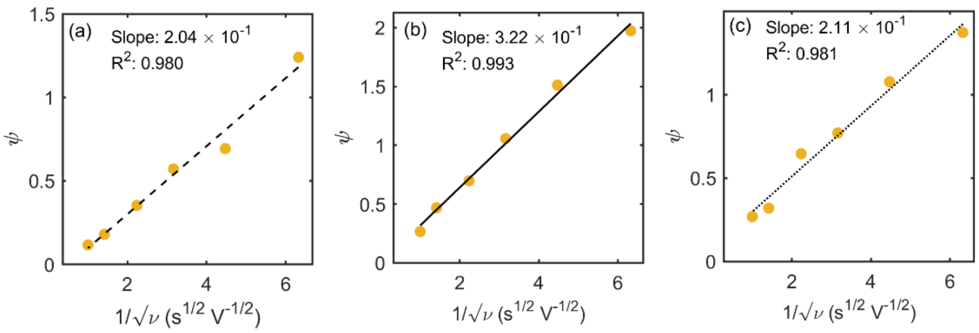

Figure S26. Plot of Nicholson dimensionless parameter (ψ) versus (ν)$^{-1/2}$ for monomeric species of a) **[(F$_2$tmpa)Cu^I]$^+$** b) **[(tmpa)Cu^I]$^+$** and c) **[(MeTFE-tmpa)Cu^I]$^+$** in DMF.

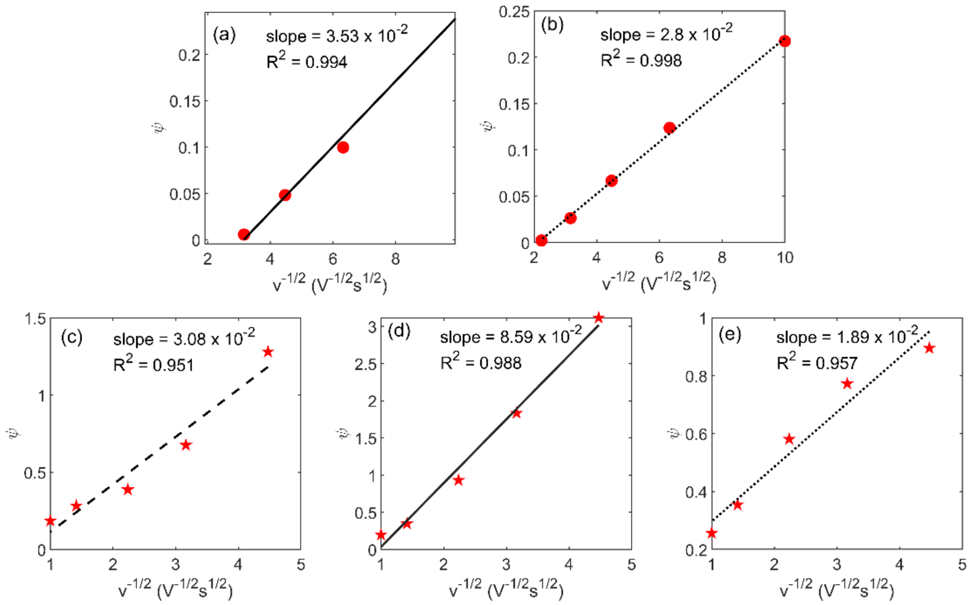

Figure S27. Plot of Nicholson dimensionless parameter (ψ) versus (ν)$^{-1/2}$ for dimeric species of c) **[(F$_2$tmpa)Cu^I]$^+$** and for monomeric and dimeric species a & d) **[(tmpa)Cu^I]$^+$** and b & e) **[(MeTFE-tmpa)Cu^I]$^+$** in MeTHF.

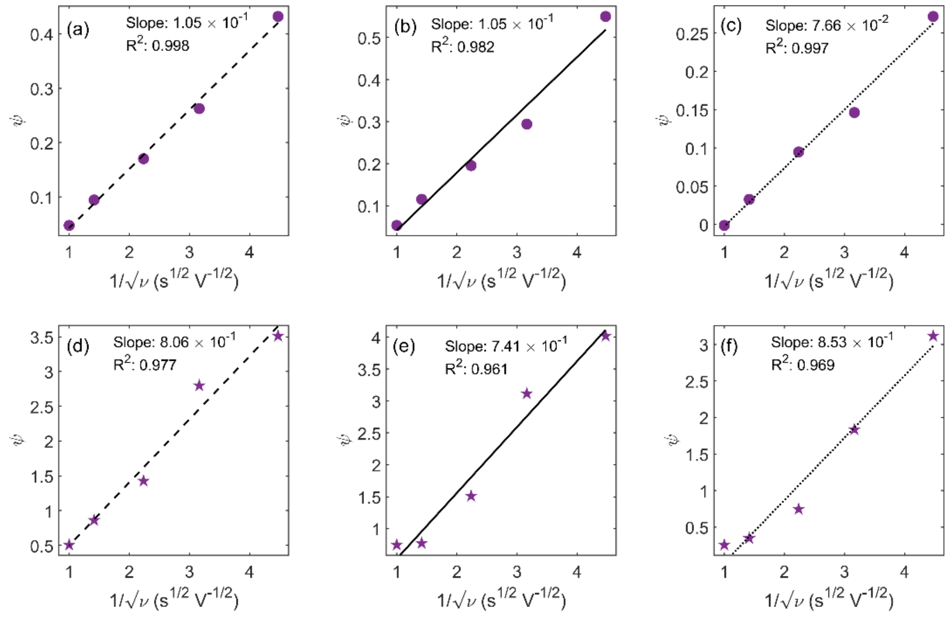

Figure S28. Plot of Nicholson dimensionless parameter (ψ) versus (ν)$^{-1/2}$ for monomeric and dimeric species of a & d) **[(F$_2$tmpa)Cu^I]$^+$** b & e) **[(tmpa)Cu^I]$^+$** and c & f) **[(MeTFE-tmpa)Cu^I]$^+$** in acetone.

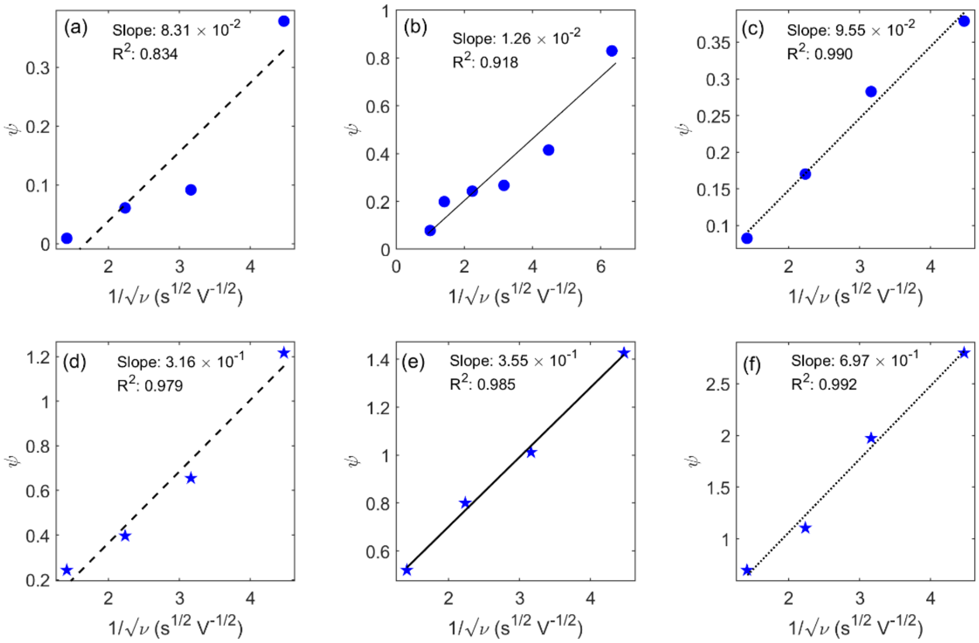

Figure S29. Plot of Nicholson dimensionless parameter (ψ) versus (ν)$^{-1/2}$ for monomeric and dimeric species of a & d) **[(F2tmpa)CuI]+** b & e) **[(tmpa)CuI]+** and c & f) **[(MeTFE-tmpa)CuI]+** in THF.

g. Kinetic modeling and determination of the dissociation rate constant for the oxidized dimer of ***[(tmpa)CuI]+*** *in acetone.*

Analysis of cyclic voltammetry data at variable scan rates revealed distinct trends in peak-current ratios. For relatively polar solvents such as acetone, DMF and MeCN, the anodic-to-cathodic peak-current ratio for the monomer decreases from unity, whereas a reciprocal trend is seen for dimer as the scan rate is decreased. This behavior indicates a chemical step occurring subsequent to electrochemical oxidation, as illustrated in Scheme S1:

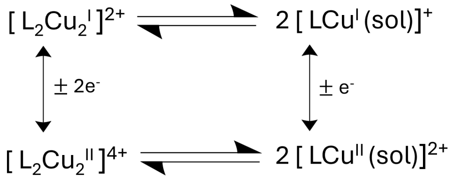

Scheme S1: Proposed ECE mechanism of copper complex redox chemistry.

We hypothesized that anodic oxidation of the dimer, $[Cu^I]_2$, initially generates the $[Cu^{II}]_2$ dimer, which subsequently undergoes first-order dissociation to yield two $[Cu^{II}]$ monomers. These monomers are reduced at the cathodic wave for the monomer couple during the reverse potential sweep. To quantify the first order rate constant for this dissociation step, we analyzed variable scan rate CV data for **[(tmpa)CuI]+** in acetone as representative example.

Using the Randles-Sevcik equation, we calculated the total concentration of $[Cu^{II}]$ species at time t:

$$[Cu^{II}]_t = [Cu^I]_0 \left(\frac{i_{pc}}{i_{pa}}\right)_{mono} \frac{\sqrt{D_{[Cu^I]}}}{\sqrt{D_{[Cu^{II}]}}}$$

Where, i_{pa} and i_{pc} are the anodic and cathodic peak current for the monomer. $[Cu^I]_0$ is the bulk concentration of Cu^I monomer whereas, $[Cu^{II}]_t$ is the total concentration of Cu^{II} monomer at time t, which represent the electrochemically generated $[Cu^{II}]$ plus $[Cu^{II}]$ generated as a result of dissociation of $[Cu^{II}]_2$. The corresponding excess monomer current thus provides a direct measure of the extent of dimer dissociation as a function of the electrochemical time window (ΔE = 1.40 V). The change in $[Cu^{II}]$ concentration is related to $[Cu^{II}]_2$ concentration as,

$$\Delta[Cu^{II}] = [Cu^{II}]_t - 1.50\,\text{mM} = 2 \times \Delta[Cu^{II}]_2$$

Thus, the concentration of Cu^{II} dimer at given time is calculated by:

$$[Cu^{II}]_{2(t)} = [Cu^{I}]_2 - \frac{\Delta[Cu^{II}]}{2}$$

The dissociation reaction follows first-order kinetics:

$$[Cu^{II}]_2 \xrightarrow{k_{diss}} 2\,[Cu^{II}]$$

$$\frac{d[Cu^{II}]_2}{dt} = -\,k_{diss}\,[Cu^{II}]_2$$

Integrating the rate law yields:

$$ln\frac{[Cu^{II}]_{2\,(t)}}{[Cu^{II}]_{2(0)}} = -k_{diss}\,t$$

A linear plot of $ln\frac{[Cu^{II}]_{2\,(t)}}{[Cu^{II}]_{2(0)}}$ versus time yields the first-order dissociation rate constant k_{diss}.

Experimental data and calculations are summarized below:

$D_{[Cu^I]}$ = 1.17 x 10^{-06} cm^2 s^{-1} $[Cu^I]$ = 1.50 mM

$D_{[CuII]}$ = 1.05 x 10^{-06} cm^2 s^{-1} $[Cu^I]_2$ = 0.24 mM

Table S6. Scan-rate-dependent decay of $[Cu^{II}]_2$ into $[Cu^{II}]$ following electrochemical oxidation of $[Cu^{I}]_2$. As the scan rate increases, the i_{pa}/i_{pc} ratio for the monomer wave increases, reflecting reduced chemical loss of the electrochemically generated of $[Cu^{II}]_2$ species.

ν / V s^{-1}	t (s)	$\frac{ipa}{ipc}$ (mon)	$[Cu^{II}]_{(t)}$ (mM)	$\Delta[Cu^{II}]$ (mM)	$[Cu^{II}]_{2(t)}$ (mM)	$[Cu^{II}]_{2(t)}$ / $[Cu^{II}]_{2(0)}$	ln $[Cu^{II}]_{2(t)}$ / $[Cu^{II}]_{2(0)}$
0.01	140	0.888	1.78	0.282	0.099	0.413	-0.885
0.025	56	0.932	1.70	0.199	0.140	0.585	-0.536
0.05	28	0.953	1.66	0.160	0.160	0.666	-0.406
0.1	16	0.963	1.64	0.144	0.168	0.700	-0.357
0.2	7	0.976	1.62	0.121	0.179	0.747	-0.292
0.5	2.8	0.994	1.59	0.093	0.193	0.806	-0.216
1	1.4	0.991	1.60	0.097	0.191	0.798	-0.226

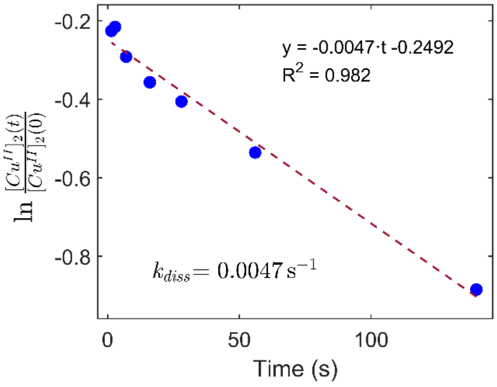

Figure S30. A first order kinetic plot describing the fraction of $[Cu^{II}]_2$ species remaining at time t for **$[(tmpa)Cu^I]^+$** in acetone. A linear fit yielded a slope of 0.0047 s^{-1} which corresponds to a rate constant for the chemical dissociation step: $[Cu^{II}]_2$ into 2 $[Cu^{II}]$.

$$k_{diss} = 0.0047\ s^{-1}$$

Half-life ($t_{1/2}$) of $[Cu^{II}]_2$ = 147 s

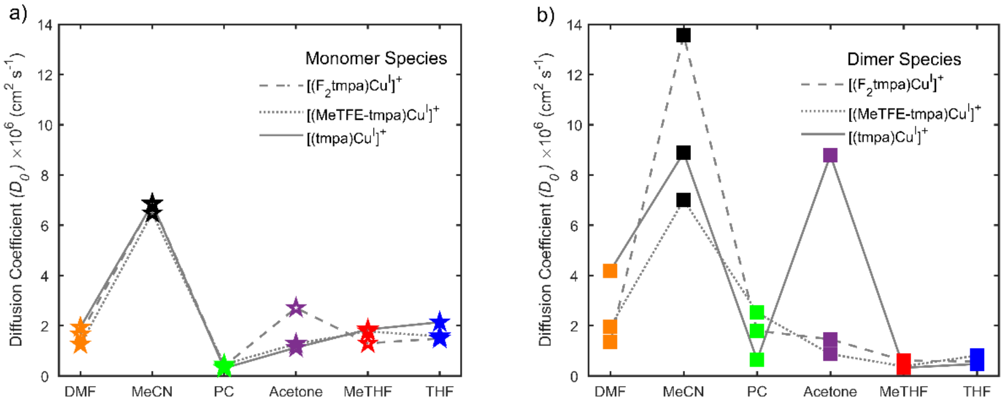

Figure S31. Diffusion coefficients of **[(tmpa)Cu^I]^+**, **[(F₂tmpa)Cu^I]^+**, and **[(MeTFE-tmpa)Cu^I]^+** in various solvents, comparing monomer (a) and dimer (b) forms.

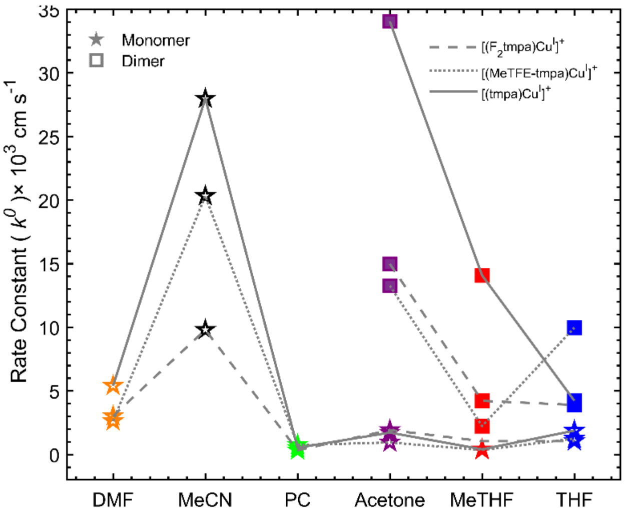

Figure S32. Plot of electron transfer rate constants (k^0) of **[(tmpa)Cu^I]^+**, **[(F₂tmpa)Cu^I]^+**, and **[(MeTFE-tmpa)Cu^I]^+** in various solvents for monomer and dimer form.

b. Effect of electrolyte properties on monomer-dimer equilibria

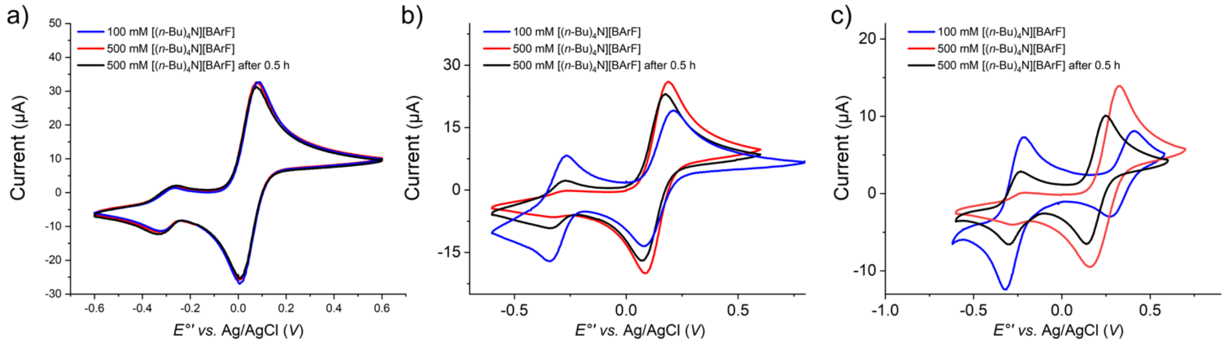

Figure S33. Effect of solvent, supporting salt concentration, and sample age on monomer-dimer equilibria in a) MeCN, b) acetone, and c) THF. Electrolyte: 2 mM **[(tmpa)Cu^I]^+** in corresponding solvent/supporting salt solution. Electrodes: 3-mm glassy carbon (working), Ag/AgCl (reference), carbon rod (counter). Scan rate: 100 mV s^{-1}.

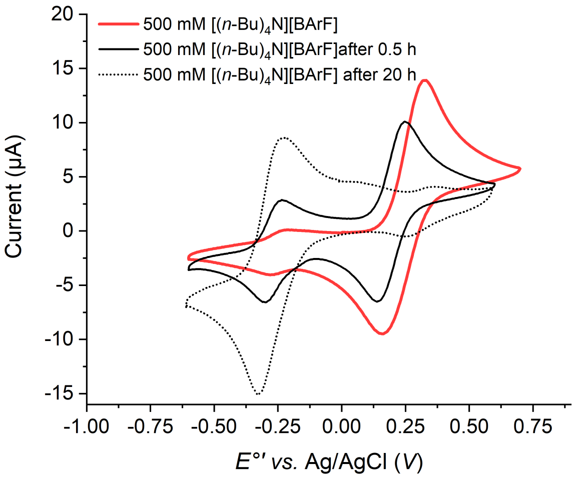

Figure S34. Cyclic voltammetry illustrating the time-dependent shift of equilibrium toward dimer form in weakly coordinating solvent like THF. Electrolyte: 2 mM **[(tmpa)Cu^{I}]$^{+}$** in 0.5 M [(*n*-Bu)$_4$N][B(C$_6$F$_5$)$_4$]. Electrodes: 3-mm glassy carbon (working), Ag/AgCl (reference), carbon rod (counter). Scan rate: 100 mV s^{-1}.

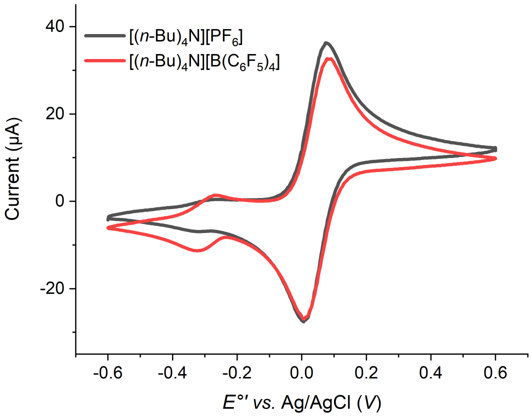

Figure S35. Effect of supporting salt on monomer-dimer equilibria in MeCN. Electrolyte: 2 mM **[(tmpa)Cu^{I}]$^{+}$** in 100 mM [(*n*-Bu)$_4$N][B(C$_6$F$_5$)$_4$] or [(*n*-Bu)$_4$N][PF$_6$]. Electrodes: 3-mm glassy carbon (working), Ag/AgCl (reference), carbon rod (counter). Scan rate: 100 mV s^{-1}.

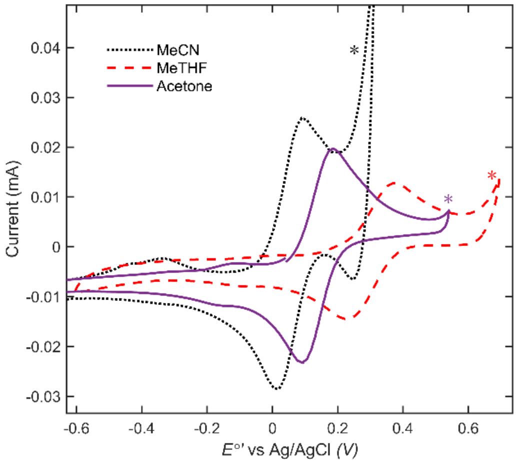

Figure S36. Bulk equilibrium speciation of of **[(tmpa)Cu^{II}]$^{2+}$** in MeCN, MeTHF, and acetone. Each voltammogram is dominated by the monomeric Cu^{II}/Cu^{I} couple, while the dimer wave appears only as a faint shoulder, confirming that the oxidized complex is largely monomeric in all three solvents. Additional features (marked with an asterisk) correspond to Ag^{+}/Ag couple arising from trace [Ag^{I}(MeCN)$_4$][B(C$_6$F$_5$)$_4$] carried over from the synthesis, whose potential matches literature values for the Ag^{+}/Ag couple in the respective solvents.[9]

i. Digital simulation of cyclic voltammograms

To validate our mechanistic assignments and the experimental diffusion and kinetic parameters, we performed digital simulations of the cyclic voltammograms in DigiElch. Using the experimentally determined diffusion coefficients, formal potentials, and rate constants, the simulated CVs at 100 mV s^{-1} reproduce both the peak currents and the peak positions for monomer/dimer waves in all the solvents studied. In MeCN, DMF, PC, and acetone, the simulated CVs overlay almost perfectly with the experimental traces, both in peak currents and monomer/dimer wave positions. In THF and MeTHF, baseline irregularities in the recorded voltammograms introduce some quantitative discrepancies, yet the overall trends in diffusion coefficient and kinetic parameters remain in agreement with the simulations. Figure S37 and Table S7 present the simulated voltammograms overlaid with the corresponding experimental CVs and list the key simulation parameters for each solvent. All values are provided below in Table S7. Where K_{eq} represents the ratio between k_f and k_b for the reversible association/dissociation between reduced monomer and dimer species. Alternatively, k_{diss} describes the dissociation of the oxidized dimer, $[Cu^{II}]_2$ into monomers, 2 $[Cu^{II}]$. Diffusion coefficients (D_0) and heterogeneous electron transfer rates (k^0) are also provided.

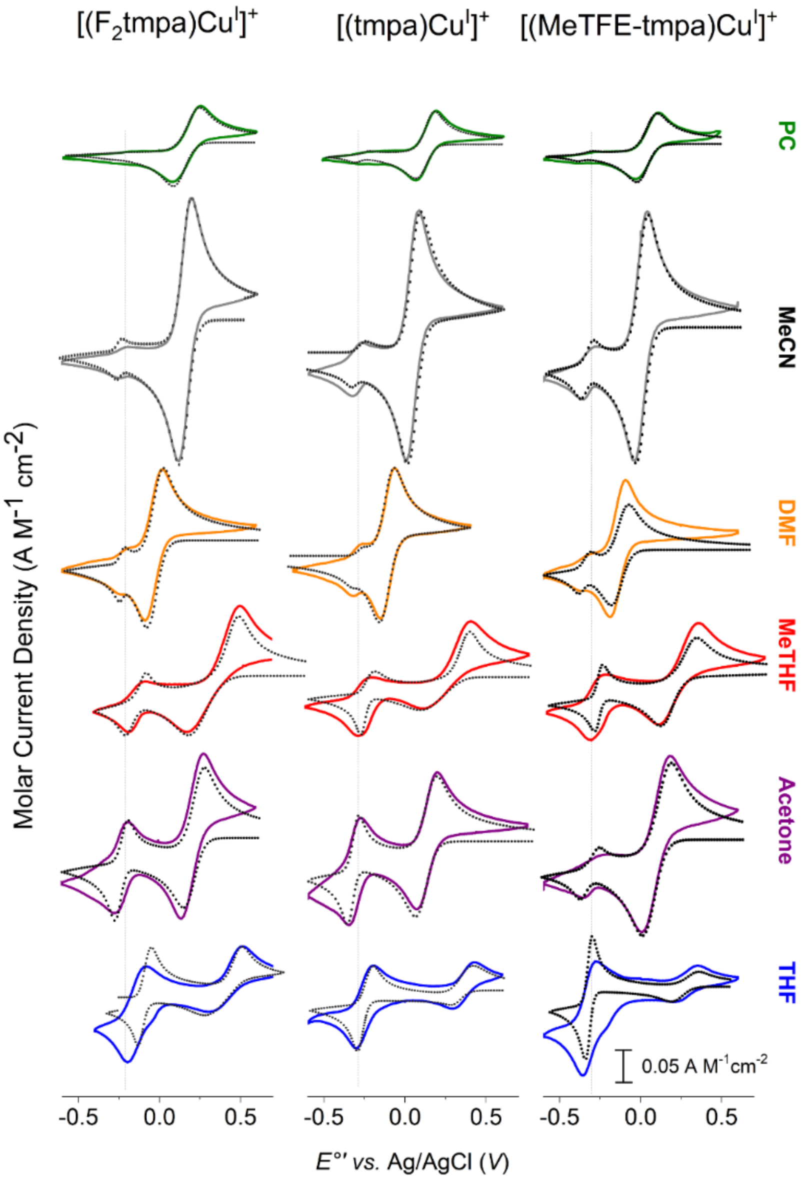

Figure S37. Overlay of experimental (solid) and DigiElch-simulated (dotted) cyclic voltammograms of copper(I) complexes in various solvents at 100 mV s^{-1}.

Table S7. Simulated electrochemical parameters for Cu(I) species across different solvents.

		$[(F_2tmpa)Cu^I]^+$		$[(tmpa)Cu^I]^+$		$[(MeTFE\text{-}tmpa)Cu^I]^+$	
		Monomer	Dimer	Monomer	Dimer	Monomer	Dimer
PC	$D_{0(\mathrm{Anodic})}$ (cm^2·s^{-1})	6.22E-07	3.55E-06	2.24E-07	3.96E-07	9.29E-70	5.05E-06
	$D_{0(\mathrm{Cathodic})}$ (cm·s^{-1})	8.22E-07	1.12E-06	3.56E-07	2.39E-07	5.15E-07	4.22E-06
	k^0 (cm s^{-1})	5.67E-04	4.3251E-04	6.33E-04	7.73E-04	9.40E-04	1.20E-03
	K_{eq} (M^{-1})	20.714		9.9558		5.05	
	k_f (M^{-1} s^{-1})	0.03565		0.10171		0.03456	
	k_b (s^{-1})	0.0017		0.0102		0.00684	
	k_{diss} (s^{-1})	0.11163		0.057285		0.0644	
MeCN	$D_{0(\mathrm{Anodic})}$ (cm^2·s^{-1})	4.55E-06	2.32E-07	8.32E-06	7.13E-05	5.75E-06	2.35E-06
	$D_{0(\mathrm{Cathodic})}$ (cm·s^{-1})	5.04E-06	5.01E-06	7.13E-05	6.98E-07	4.73E-06	1.22E-05
	k^0 (cm s^{-1})	0.01981	0.014544	0.023491	0.014427	0.0139	0.028
	K_{eq} (M^{-1})	5.2666		20.97		10.01	
	k_f (M^{-1} s^{-1})	0.00010542		0.085819		0.001	
	k_b (s^{-1})	2.00E-05		0.00409		0.0001	
	k_{diss} (s^{-1})	0.00014348		0.053426		0.00127	
DMF	$D_{0(\mathrm{Anodic})}$ (cm^2·s^{-1})	2.22E-06	3.04E-07	3.01E-06	7.05E-06	1.75E-06	1.90E-06
	$D_{0(\mathrm{Cathodic})}$ (cm·s^{-1})	2.97E-06	3.29E-06	3.69E-06	1.46E-05	1.92E-06	9.00E-07
	k^0 (cm s^{-1})	0.0040478	0.011936	0.0095147	0.0069651	0.0026	0.0013
	K_{eq} (M^{-1})	7.4064		12.147		1.25	
	k_f (M^{-1} s^{-1})	0.034639		0.0028718		0.00518	
	k_b (s^{-1})	0.0046		2.30E-05		0.00415	
	k_{diss} (s^{-1})	0.0099791		0.013853		0.008	
MeTHF	$D_{0(\mathrm{Anodic})}$ (cm^2·s^{-1})	7.59E-06	8.89E-06	4.77E-06	2.49E-05	3.74E-06	2.10E-06
	$D_{0(\mathrm{Cathodic})}$ (cm·s^{-1})	2.75E-06	7.28E-06	1.38E-06	4.73E-06	2.85E-06	1.20E-06
	k^0 (cm s^{-1})	0.00037898	0.0010563	0.00035287	0.0040348	0.000501	0.0029
	K_{eq} (M^{-1})	339.28		510.05		1029	
	k_f (M^{-1} s^{-1})	0.00017365		0.050076		0.00152	
	k_b (s^{-1})	5.11E-7		9.10E-05		1.47716E-06	
	k_{diss} (s^{-1})	0.056895		0.080475		0.04096	
Acetone	$D_{0(\mathrm{Anodic})}$ (cm^2·s^{-1})	2.05E-06	2.46E-06	3.46E-06	1.46E-05	3.74E-06	3.98E-06
	$D_{0(\mathrm{Cathodic})}$ (cm·s^{-1})	3.20E-06	1.40E-05	2.40E-06	4.22E-05	3.17E-06	1.80E-06
	k^0 (cm s^{-1})	0.0021347	0.0025519	0.0020782	0.0053824	0.001	0.0085
	K_{eq} (M^{-1})	498.24		469		107.47	
	k_f (M^{-1} s^{-1})	0.0090948		0.0021683		0.00203	
	k_b (s^{-1})	1.82E-05		4.96E-03		1.889E-05	
	k_{diss} (s^{-1})	0.074864		0.091554		0.0286	
THF	$D_{0(\mathrm{Anodic})}$ (cm^2·s^{-1})	2.32E-05	4.05E-07	9.85E-08	1.69E-06	2.17E-06	2.00E-06
	$D_{0(\mathrm{Cathodic})}$ (cm·s^{-1})	2.60E-05	2.09E-07	3.09E-07	7.76E-07	8.43E-06	1.00E-06
	k^0 (cm s^{-1})	0.001695	0.00056995	0.00045252	0.0008536	0.0011	0.00432
	K_{eq} (M^{-1})	12847		1.03E+05		17261	
	k_f (M^{-1} s^{-1})	0.0050143		4.6738		0.0305	
	k_b (s^{-1})	3.90E-07		4.54E-05		1.7669E-06	
	k_{diss} (s^{-1})	0.028001		0.081288		0.011	

3. Variable-temperature NMR in varying solvents

Unless otherwise specified, all sample preparations were done under an N_2 atmosphere inside a glovebox to prevent the complexes from getting oxidized, as reported earlier by our group.[1] To prepare the NMR samples, 10 mg (12 mM) of each **[(F$_2$tmpa)Cu^I][B(C$_6$F$_5$)$_4$]**, **[(tmpa)Cu^I][B(C$_6$F$_5$)$_4$]**, or **[(MeTFE-tmpa)Cu^I][B(C$_6$F$_5$)$_4$]** were weighed in three different vials, dissolved in THF-*d$_8$* (800 μl), and transferred into three different NMR tubes. The NMR tubes were then sealed using a rubber septum prior to removal from the glovebox. To ensure uniform cooling, each sample was allowed to equilibrate at the desired temperature for at least 5 minutes prior to data collection. The NMR spectra of the above samples in MeCN-*d$_3$*, DMF-*d$_7$*, and acetone-*d$_6$* were recorded following the same method described above.

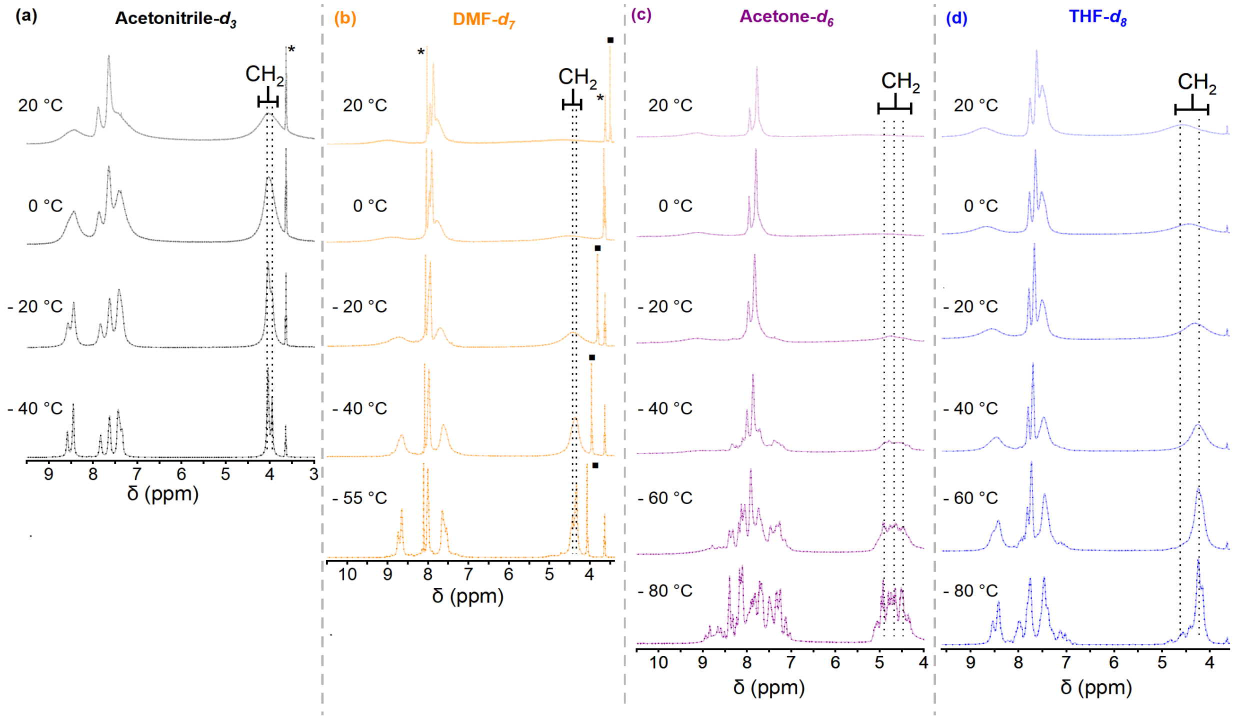

Figure S38. Part of the variable-temperature (12 mM, 20 °C to –80 °C) ^{1}H NMR (400 MHz) spectra of the **[(F$_2$tmpa)Cu^I]$^+$** complex in (a) MeCN-*d$_3$*, (b) DMF-*d$_7$*, (c) acetone-*d$_6$*, and (d) THF-*d$_8$*. The well-resolved peak in the aliphatic and aromatic region at lower temperatures indicates different proton environments for both aliphatic and aromatic protons, suggesting the dominance of dimeric structure. Peaks marked with * and ▪ correspond to the solvent peak and H_2O peak, respectively.

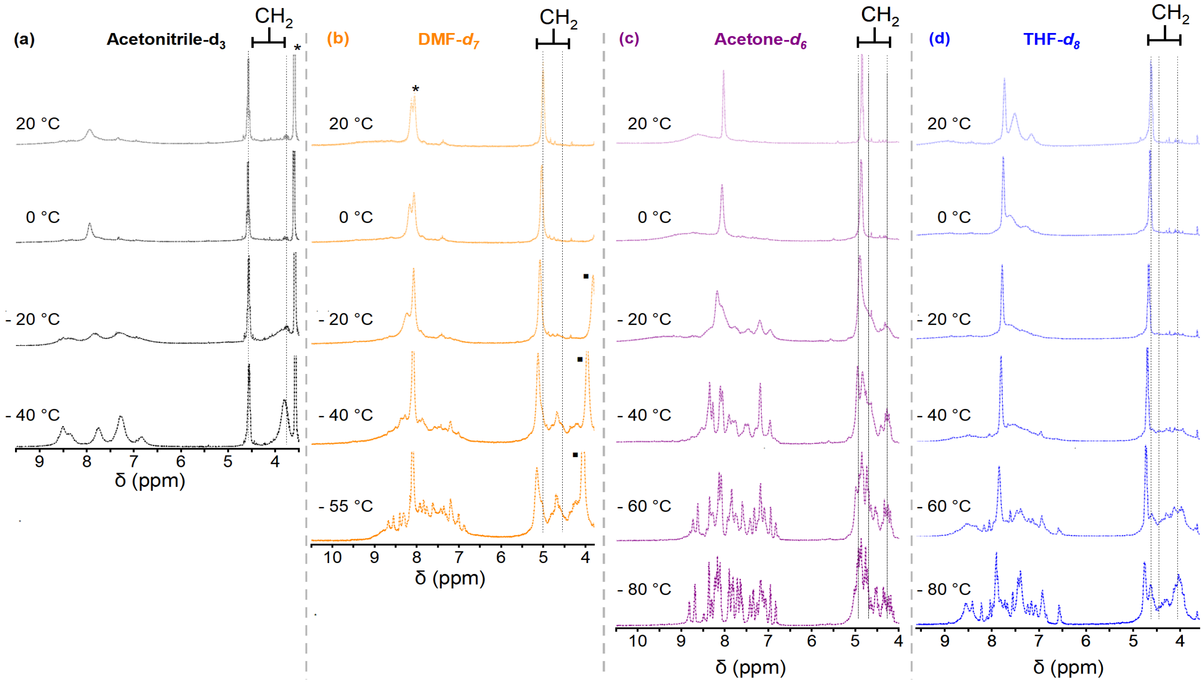

Figure S39. Part of the variable-temperature (12 mM, 20 °C to –80 °C) ^{1}H NMR (400 MHz) spectra of the **$[(MeTFE\text{-}tmpa)Cu^I]^+$** complex in (a) MeCN-*d*$_3$, (b) DMF-*d*$_7$, (c) acetone-*d*$_6$, and (d) THF-*d*$_8$. The appearance of well-resolved peaks in the aliphatic and aromatic region at lower temperatures indicates different proton environments for both aliphatic and aromatic protons, suggesting the dominance of dimeric structure. Peaks marked with * and ▪ corresponds to the solvent peak and H_2O peak, respectively.

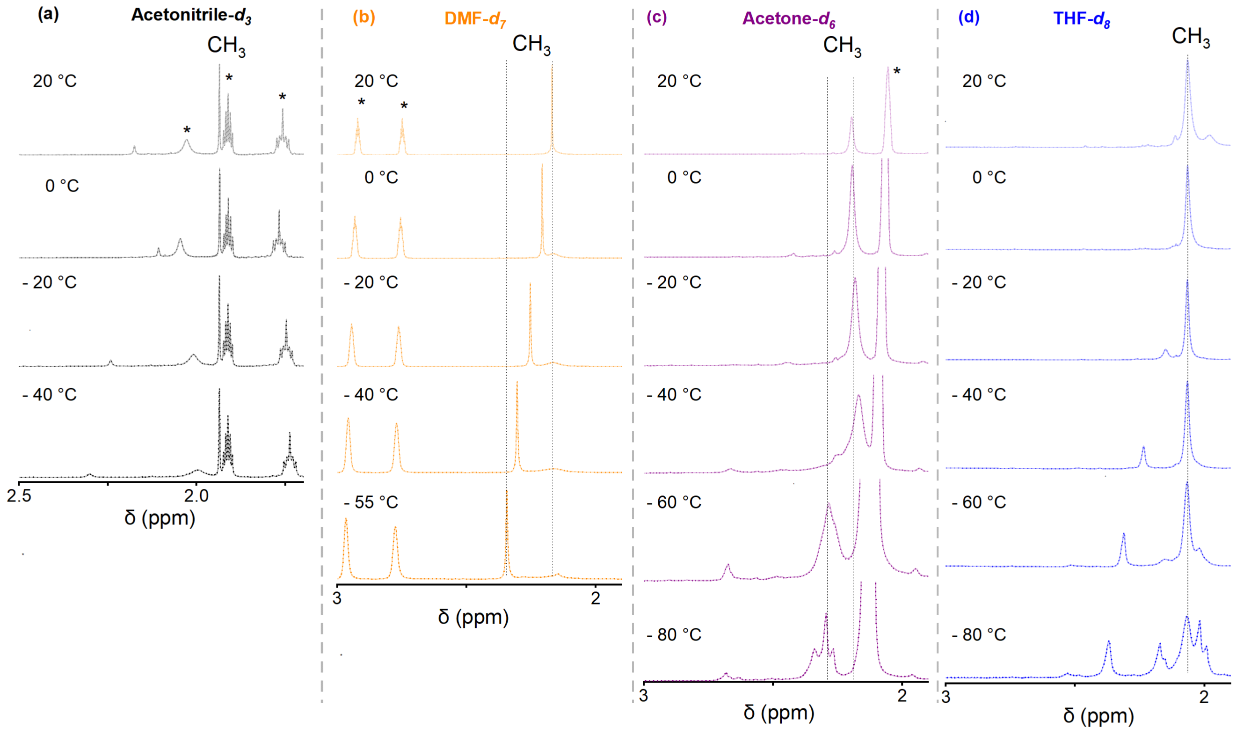

Figure S40. Specific aliphatic part of the variable-temperature (12 mM, 20 °C to –80 °C) ^{1}H NMR (400 MHz) spectra of the **$[(MeTFE\text{-}tmpa)Cu^I]^+$** complex in (a) MeCN-*d*$_3$, (b) DMF-*d*$_7$, (c) acetone-*d*$_6$, and (d) THF-*d*$_8$. In the dimeric structure, each copper ion is ligated by two pyridyl arm, one bridging pyridyl arm, and one amine nitrogen. From the data obtained at –80 °C in THF-*d*$_8$, it appears that ⅔ of methyl bound pyridyl arms could coordinate to the copper while 1/3 of methyl bound pyridyl arms bridge to the second copper leading to a 2:1 ratio proton signal. Additional splittings observed are likely due to the difference in chemical environments or exchange couplings. Peaks marked with * and ▪ corresponds to the solvent peak and H_2O peak, respectively.

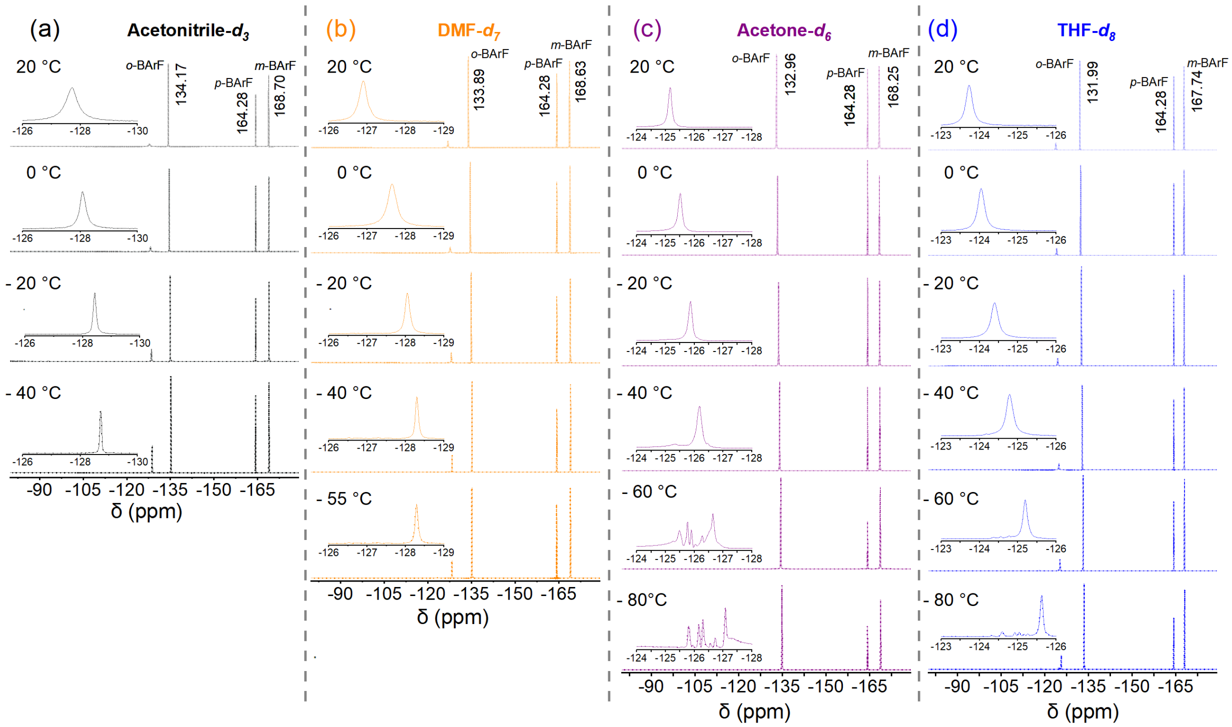

Figure S41. Variable-temperature (12 mM,20 °C to –80 °C) ^{19}F NMR (376 MHz) spectra of the **[(F$_2$tmpa)Cu^I][B(C$_6$F$_5$)$_4$]** complex in (a) MeCN-*d$_3$*, (b) DMF-*d$_7$*, (c) acetone-*d$_6$*, and (d) THF-*d$_8$*. Inset: Expanded NMR Signal of the fluorine directly attached to the pyridyl arm. The splitting of the peak of the pyridyl fluorine at lower temperatures reflects the different environments of the fluorine, strongly suggesting the existence of a dimeric structure.

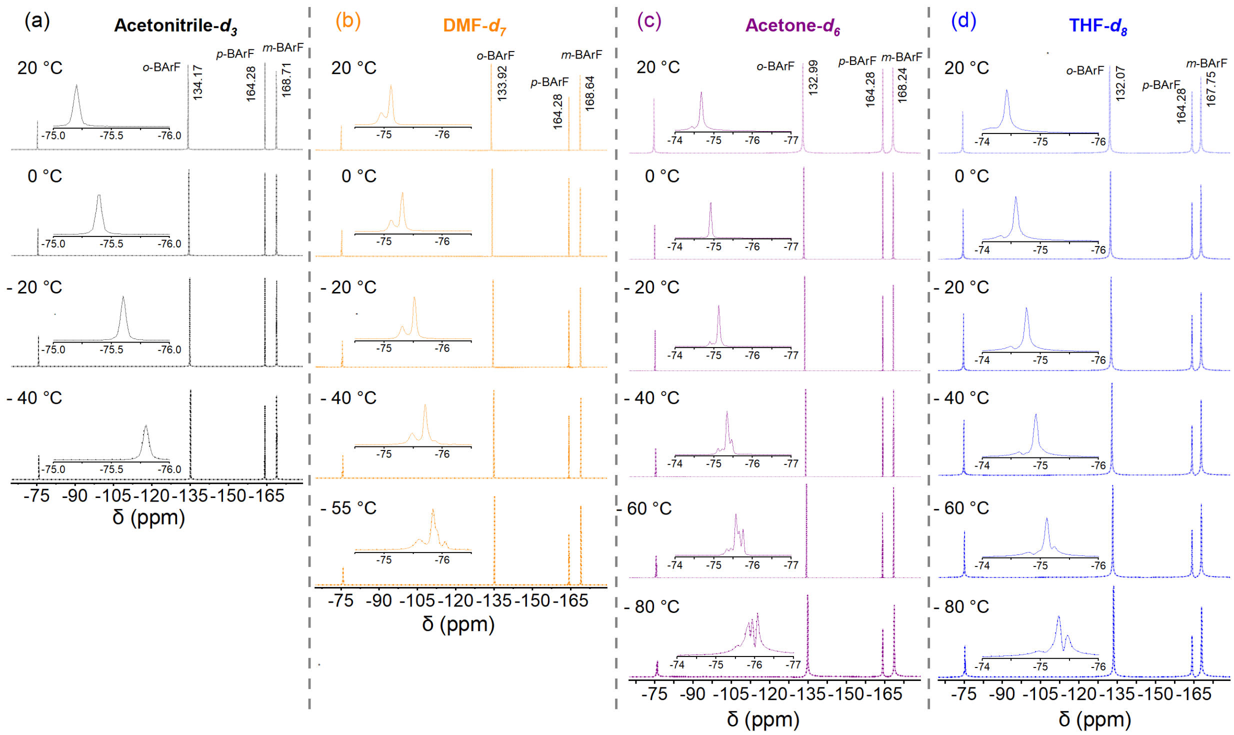

Figure S42. Variable temperature (12 mM, 20 °C to –80 °C) ^{19}F NMR (376 MHz) spectra of the **[(MeTFE-tmpa)Cu^I][B(C$_6$F$_5$)$_4$]** complex in (a) MeCN-*d$_3$*, (b) DMF-*d$_7$*, (c) acetone-*d$_6$*, and (d) THF-*d$_8$*. Inset: Expanded NMR signal of the -CF$_3$ fluorine of trifluoroethoxy moiety attached to the pyridyl arm. The splitting of the -CF$_3$ fluorine peak at low temperatures reflects the different environment of the fluorine and suggests the existence of a dimeric structure.

a. Thermodynamic analysis of dimerization from VT-NMR data

The equilibrium constant (K_{eq}) for dimerization of **[(F$_2$tmpa)Cu^I][B(C$_6$F$_5$)$_4$]** and **[(MeTFE-tmpa)Cu^I][B(C$_6$F$_5$)$_4$]** were determined using variable-temperature (VT) ^{19}FNMR spectroscopy. The analysis assumes that the complex exists predominantly as a monomer in MeCN-*d$_3$* at room temperature and shifts to a predominantly dimeric form in acetone-*d$_6$* at –80 °C. Acetone-*d$_6$* was selected as the solvent for dimer as the ^{1}H NMR spectra exhibited well-resolved splitting of aromatic resonances, indicative of increased molecular asymmetry associated with predominant dimer formation. The chemical shifts corresponding to the monomeric (δ_m) and dimeric (δ_d) species were used to estimate the mole fractions (f) of each species. These mole fractions were then used to determine the equilibrium constant (K_{eq}) and Gibbs free energy (ΔG) using equation 7.[10] Given that chemical shifts are sensitive to solvent and temperature, a significant source of error can arise when comparing data across different conditions. To minimize this, the chemical shift of the ortho-F atom of the [B(C$_6$F$_5$)$_4$]$^-$ anion or [BArF]$^-$ (*o*-BArF) at -134.2 ppm for **[(F$_2$tmpa)Cu^I][B(C$_6$F$_5$)$_4$]** and *meta*-F atom of [BArF]$^-$ (*m*-BArF) at -168.3 ppm for **[(MeTFE-tmpa)Cu^I][B(C$_6$F$_5$)$_4$]**, both in MeCN-*d$_3$*, were used as an internal reference. The *o*- and *m*-F atom were selected based on the position of F on the pyridine arms of the ligand to best represent those positions. All NMR shifts in both MeCN-*d$_3$*, and acetone-*d$_6$* were referenced to this signal to ensure consistency and reduce systematic error. The standard thermodynamic parameters were calculated following the Van't Hoff equation 8.

$$f_m = \frac{\delta_d - \delta_{obsd}}{\delta_d - \delta_m} \qquad f_d = \frac{\delta_{obsd} - \delta_m}{\delta_d - \delta_m}$$

f_d is the mole fraction of dimer, f_m is mole fraction of the monomer. δ_d is chemical shift of dimer (acetone-*d$_6$* at –80 °C), δ_m is chemical shift of monomer (MeCN-*d$_3$* at 20 °C), δ_{obsd} is the actual chemical shift observed after referencing.

$$K_{eq} = \left(\frac{f_d}{2[A]_0 f_m^2}\right) \quad \ldots\ldots\ldots\ldots\ldots\ldots\ldots\ldots \quad \text{(Eq. 7)}$$

$$\Delta G = -RT\ln K_{eq}$$

$$\ln(K_{eq}) = -\frac{\Delta H^0}{R}\frac{1}{T} + \frac{\Delta S^0}{R} \quad \ldots\ldots\ldots\ldots\ldots\ldots\ldots\ldots \quad \text{(Eq. 8)}$$

$$\Delta G^0 = \Delta H^0 - T\Delta S^0$$

Where $[A]_0$ is the total concentration of monomer and dimer combined, ΔG^0 is standard Gibbs free energy, ΔH^0 is standard change in enthalpy, ΔS^0 is standard change in entropy, and R is gas constant. At low temperatures, the ^{19}F NMR signal splits into multiple peaks due to increased molecular asymmetry. Therefore, δ_d or δ_{obsd} was taken as the average chemical shift of the observed peak involved. Based on this analysis, at 20 °C in acetone-*d$_6$*, the equilibrium mixture consists of approximately 68% dimer and 32% monomer in **[(F$_2$tmpa)Cu^I][B(C$_6$F$_5$)$_4$]**, corresponding to an K_{eq} of 2.77 × 10^2 M^{-1}. At –60 °C, the equilibrium shifts further toward the dimer, with a dimer-to-monomer ratio of 0.97:0.03 and an estimated K_{eq} of 3.82 × 10^4 M^{-1}. Complete details of the chemical shifts, mole fractions, K_{eq}, and other thermodynamic parameters are provided in Tables S8-S10.

Table S8. Table for the estimation of fraction of dimeric and monomeric species and K_{eq} in acetone-*d$_6$*

**[(F$_2$tmpa)Cu^I][B(C$_6$F$_5$)$_4$)]***					
Temperature (°C)	Observed Shift (δ_{obsd})	f_m	f_d	K_{eq} (M^{-1})	ΔG (kcal•mol^{-1})
20	-126.36	0.32	0.68	2.77 × 10^2	-3.27
0	-126.36	0.32	0.68	2.77 × 10^2	-3.05
-20	-126.34	0.31	0.69	2.99 × 10^2	-2.86
-40	-125.87	0.08	0.92	6.85 × 10^3	-4.09
-60	-125.785	0.03	0.97	3.82 × 10^4	-4.46
**[(MeTFE-tmpa)Cu^I][B(C$_6$F$_5$)$_4$)]****					
Temperature (°C)	Observed Shift (δ_{obsd})	f_m	f_d	K_{eq} (M^{-1})	ΔG (kcal•mol^{-1})
0	-74.87	0.84	0.16	9.03 × 10^0	-1.19
-20	-75	0.66	0.34	3.21 × 10^1	-1.74
-40	-75.13	0.48	0.52	9.47 × 10^1	-2.10
-60	-75.32	0.21	0.79	7.36 × 10^2	-2.79

*The chemical shift for the monomer and dimer of **[(F$_2$tmpa)Cu^I][B(C$_6$F$_5$)$_4$)]** were estimated as δ_m = -127.72 ppm in MeCN-*d$_3$* and δ_d= -125.72 ppm in acetone-*d$_6$*. **The chemical shift for the monomer and dimer of **[(MeTFE-tmpa)Cu^I][B(C$_6$F$_5$)$_4$)]** were estimated as δ_m = -74.76 ppm and δ_d = -75.47 in acetone-*d$_6$*.

Table S9. Table for the estimation of fraction of dimeric and monomeric species and K_{eq} in DMF-d_7

**[(F$_2$tmpa)Cu^I][B(C$_6$F$_5$)$_4$)]***					
Temperature (°C)	Observed Shift (δ_{obsd})	f_m	f_d	K_{eq} (M^{-1})	ΔG (kcal•mol^{-1})
20	-127.18	0.72	0.28	2.26×10^1	-1.82
0	-127.28	0.77	0.23	1.63×10^1	-1.52
-20	-124.36	0.80	0.20	1.27×10^1	-1.28
-40	-123.58	0.36	0.64	1.99×10^2	-2.45
-55	-123.58	0.36	0.64	1.99×10^2	-2.29
**[(MeTFE-tmpa)Cu^I][B(C$_6$F$_5$)$_4$)]****					
Temperature (°C)	Observed Shift (δ_{obsd})	f_m	f_d	K_{eq} (M^{-1})	ΔG (kcal•mol^{-1})
20	-74.81	0.97	0.03	3.39×10^0	-0.13
0	-74.91	0.79	0.21	1.41×10^1	-1.44
-20	-75	0.66	0.34	3.21×10^1	-1.74
-40	-75.1	0.52	0.48	7.35×10^1	-1.99
-55	-75.16	0.44	0.56	1.23×10^2	-2.08

*The chemical shift for the monomer and dimer of **[(F$_2$tmpa)Cu^I][B(C$_6$F$_5$)$_4$)]** were estimated as δ_m = -127.72 ppm in MeCN-d_3 and δ_d = -125.72 ppm in acetone-d_6. **The chemical shift for the monomer and dimer of **[(MeTFE-tmpa)Cu^I][B(C$_6$F$_5$)$_4$)]** were estimated as δ_m = -74.76 ppm and δ_d = -75.47 in acetone-d_6.

Table S10. Thermodynamic parameters for dimerization of **[(F$_2$tmpa)Cu^I][B(C$_6$F$_5$)$_4$)]** and **[(MeTFE-tmpa)Cu^I][B(C$_6$F$_5$)$_4$]** in acetone and DMF.

		ΔG^0 (kcal•mol^{-1})	ΔH^0 (kcal•mol^{-1})	ΔS^0 (cal•K^{-1}•mol^{-1})
Acetone-d_6	**[(F$_2$tmpa)Cu^I][B(C$_6$F$_5$)$_4$)]**	-2.072 ± 2.7	-10.45 ± 1.20	-28.12 ± 5.01
	**[(MeTFE-tmpa)Cu^I][B(C$_6$F$_5$)$_4$)]**	-0.52 ± 0.68	-8.29 ± 0.30	-26.07 ± 1.27
DMF-d_7	**[(F$_2$tmpa)Cu^I][B(C$_6$F$_5$)$_4$)]**	-2.072 ± 3.05	-12.26 ± 1.42	-31.71 ± 5.46
	**[(MeTFE-tmpa)Cu^I][B(C$_6$F$_5$)$_4$)]**	-0.86 ± 0.96	-5.83 ± 0.44	-16.69 ± 1.76

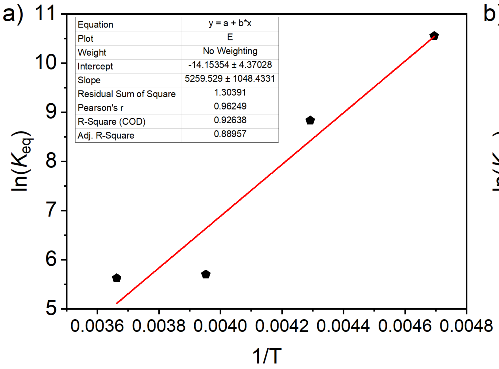

Figure S43. Van't Hoff analysis used to determine the thermodynamic parameters for the dimerization of **[(F$_2$tmpa)Cu^I][B(C$_6$F$_5$)$_4$]** in a) acetone-d_6 b) DMF-d_7. The K_{eq} was estimated from VT-NMR ^{19}F NMR data using equation 7.

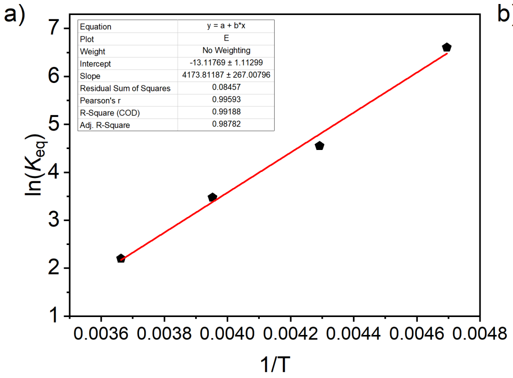

Figure S44. Van't Hoff analysis used to determine the thermodynamic parameters for the dimerization of **[(MeTFE-tmpa)Cu^I][B(C$_6$F$_5$)$_4$]** in a) acetone-d_6 b) DMF-d_7. The K_{eq} was estimated from VT-NMR ^{19}F NMR data using equation 7.

4. Single crystal X-ray diffraction

Single crystals of **$[(tmpa)_2Cu^I_2][PF_6]_2$** and **$[(F_2tmpa)_2Cu^I_2][B(C_6F_5)_4)]_2$** were obtained by slow liquid diffusion in 5 mm glass tubes using THF solutions (10 mM) layered with hexanes at RT inside the glovebox. Crystals suitable for X-ray analysis were typically obtained in a few days or weeks.

For **$[(tmpa)_2Cu^I_2][PF_6]_2$**, all reflection intensities were measured at 110(2) K using a SuperNova diffractometer (equipped with Atlas detector) with Cu-*K*α radiation (λ = 1.54178 Å). Data collection, refinement, and reduction were performed using the CrysAlisPro software (version 1.171.36.24, Agilent Technologies, 2012). Structure determination was completed using SHELXT-2013 and refined on F^2 by full-matrix least-squares methods with the SHELXL-2013 program package.[11] An analytical absorption correction based on Gaussian integration was applied using a multifaceted crystal model in CrysAlisPro. For **$[(F_2tmpa)_2Cu^I_2][B(C_6F_5)_4)]_2$**, all reflection intensities were measured at 100(2) K using a Gemini R diffractometer (with an Atlas detector) and Mo-*K*α radiation (λ = 0.71073 Å) under the CrysAlisPro software (version 1.171.38.43f, Agilent Technologies, 2015). A later version (CrysAlisPro 1.171.40.53, Agilent Technologies, 2019) was used to refine the cell dimensions and perform data reduction. The structure was determined using the SHELXT-2018/2 program and refined on F^2 by full-matrix least-squares methods with the SHELXL-2018/3 program package.[12] A numerical absorption correction based on Gaussian integration was applied using a multifaceted crystal model in CrysAlisPro. The data collection temperature for both samples was maintained using the Cryojet system (Oxford Instruments, Abingdon, UK). For the final refinement, contributions from a partial occupancy/disordered tetrahydrofuran molecule in the crystal lattice of **$[(F_2tmpa)_2Cu^I_2][B(C_6F_5)_4)]_2$** were removed using SQUEEZE. All details of the SQUEEZE refinement are provided in the final CIF file. The SUMP command in SHELXL was employed to fix the occupancy factors of the fluorine atoms on the pyridyl ring to two for this complex.

Geometry indices around the Cu center suggest a flattened tetrahedral geometry. Geometry indices can be used to describe the geometry adopted by four- or five-coordinate complexes quantitatively. The geometric indices here (τ_4 and τ'_4) were calculated based on previous literature using the following equations:[13, 14]

$$\tau_4 = \left(\frac{360 - (\alpha + \beta)}{141}\right)$$

$$\tau_4' = \frac{\beta - \alpha}{250.5} + \frac{180 - \beta}{70.5}$$

where α and β (where β > α) are the two largest angles among the six angles formed in a four-coordinate complex by the four M-L bonds. For both the structures, non-hydrogen atoms were refined anisotropically, while hydrogen atoms were placed at calculated positions using the instructions AFIX 23 or AFIX 43 with isotropic displacement parameters having values 1.2 times U_{eq} of the linked carbon atoms. Supplementary crystallographic data for this article is available at the Cambridge Crystallographic Data Center (CCDC No. 2357378 and 2357379) and can be obtained free of charge via https://www.ccdc.cam.ac.uk/data_request/cif.

Table S11. Crystallographic data and data collection parameters for **$[(tmpa)_2Cu^I_2][PF_6]_2$** and **$[(F_2tmpa)_2Cu^I_2][B(C_6F_5)_4]_2$**

	**$[(tmpa)_2Cu^I_2][PF_6]_2$**	**$[(F_2tmpa)_2Cu^I_2][B(C_6F_5)_4]_2$**
Empirical formula	$C_{44}H_{52}N_8O_2Cu_2P_2F_{12}$	$C_{87}H_{39}B_2Cu_2F_{44}N_8$
Formula weight	1141.95	2180.96
Temperature/K	110(2)	100(2)
Crystal system	Triclinic	triclinic
Space group	P-1	P-1
a/Å	10.1000(3)	12.6129(5)
b/Å	13.3100(4)	15.5670(7)
c/Å	18.6048(6)	23.4123(11)
α/°	74.879(3)	98.231(4)
β/°	86.436(3)	94.942(4)

γ/°	77.343(3)	113.269(4)
Volume/Å^3	2355.76(13)	4127.7(3)
Z	2	2
ρ_{calc}/g cm^{-3}	1.610	1.755
μ/mm^{-1}	2.603	0.670
F(000)	1168.0	2162.0
Crystal size/mm^3	0.42 × 0.34 × 0.1	0.269 × 0.143 × 0.059
Radiation (Å)	CuKα (λ = 1.54178)	MoKα (λ = 0.71073)
2Θ range for data collection/°	7.038 to 143.788	3.544 to 50.996
Index ranges	-12 ≤ h ≤ 12, -16 ≤ k ≤ 16, -22 ≤ l ≤ 22	-15 ≤ h ≤ 15, -18 ≤ k ≤ 18, -28 ≤ l ≤ 28
Reflections collected	31800	67138
Independent reflections	9265 [R_{int} = 0.0243, R_{sigma} = 0.0200]	15348 [R_{int} = 0.1004, R_{sigma} = 0.1119]
Data/restraints/parameters	9265/314/723	15348/2/1313
Goodness-of-fit on F^2	1.024	0.997
Final R indexes [I>=2σ (I)]	R_1 = 0.0289, wR_2 = 0.0740	R_1 = 0.0545, wR_2 = 0.0945
Final R indexes [all data]	R_1 = 0.0316, wR_2 = 0.0758	R_1 = 0.1152, wR_2 = 0.1146
Largest diff. peak/hole/e Å^{-3}	0.43/-0.37	0.43/-0.53

Table S12. Selected bond lengths (Å) and bond angles (°) for **$[(tmpa)_2Cu^I_2][PF_6]_2$** and **$[(F_2tmpa)_2Cu^I_2][B(C_6F_5)_4]_2$**.

$[(tmpa)_2Cu^I_2][PF_6]_2$		$[(F_2tmpa)_2Cu^I_2][B(C_6F_5)_4]_2$	
Cu1–N1	1.9854(13)	Cu1–N1	1.995(3)
Cu1–N2	2.2504(13)	Cu1–N2	2.240(3)
Cu1–N3	2.1704(14)	Cu1–N3	2.183(3)
Cu1–N8	1.9511(13)	Cu1–N8	1.948(3)
Cu2–N4	1.9582(13)	Cu2–N4	1.971(3)
Cu2–N5	2.2101(14)	Cu2–N5	2.128(3)
Cu2–N6	2.2716(13)	Cu2–N6	2.237(3)
Cu2–N7	1.9788(13)	Cu2–N7	1.994(3)
N1–Cu1–N2	82.59(5)	N1–Cu1–N2	83.30(12)
N1–Cu1–N3	94.46(5)	N1–Cu1–N3	91.98(12)
N3–Cu1–N2	78.41(5)	N3–Cu1–N2	79.06(12)
N8–Cu1–N1	154.34(6)	N8–Cu1–N1	148.56(13)
N8–Cu1–N2	120.04(5)	N8–Cu1–N2	126.86(12)
N8–Cu1–N3	101.56(5)	N8–Cu1–N3	101.63(13)
N4–Cu2–N5	105.33(5)	N4–Cu2–N5	102.99(13)
N4–Cu2–N6	122.21(5)	N4–Cu2–N6	119.39(12)
N4–Cu2–N7	147.25(6)	N4–Cu2–N7	145.80(14)
N5–Cu2–N6	76.57(5)	N5–Cu2–N6	76.98(12)
N7–Cu2–N5	101.41(5)	N7–Cu2–N5	106.87(13)
N7–Cu2–N6	82.10(5)	N7–Cu2–N6	83.78(13)

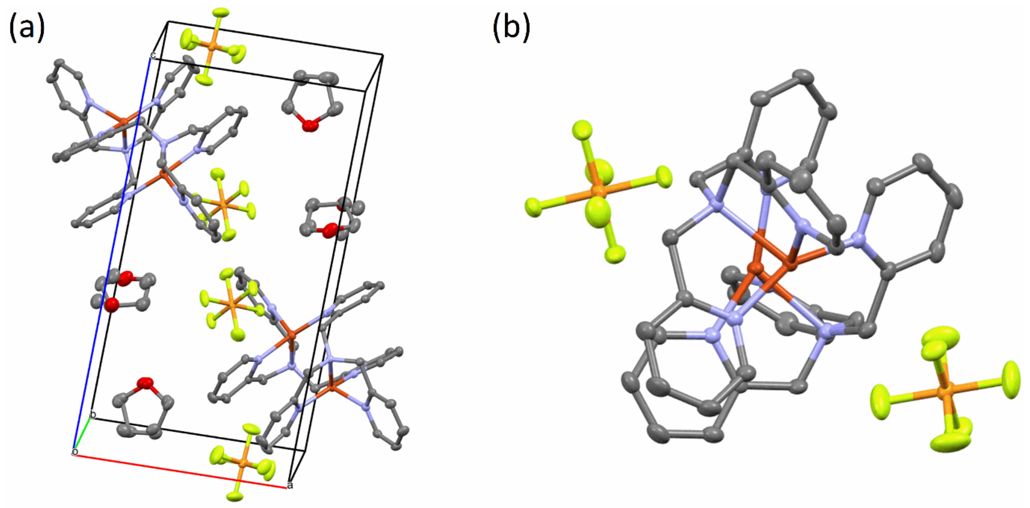

Figure S45. Diagram illustrating the (a) molecular packing of **[(tmpa)$_2$Cu$^I_2$][PF$_6$]$_2$** and (b) displacement ellipsoid plot (50% probability level, top view from Cu1) of **[(tmpa)$_2$Cu$^I_2$][PF$_6$]$_2$** at 110(2) K. A view along the Cu1 (front) and Cu2 (back) axis is shown here. All hydrogens have been omitted for clarity.

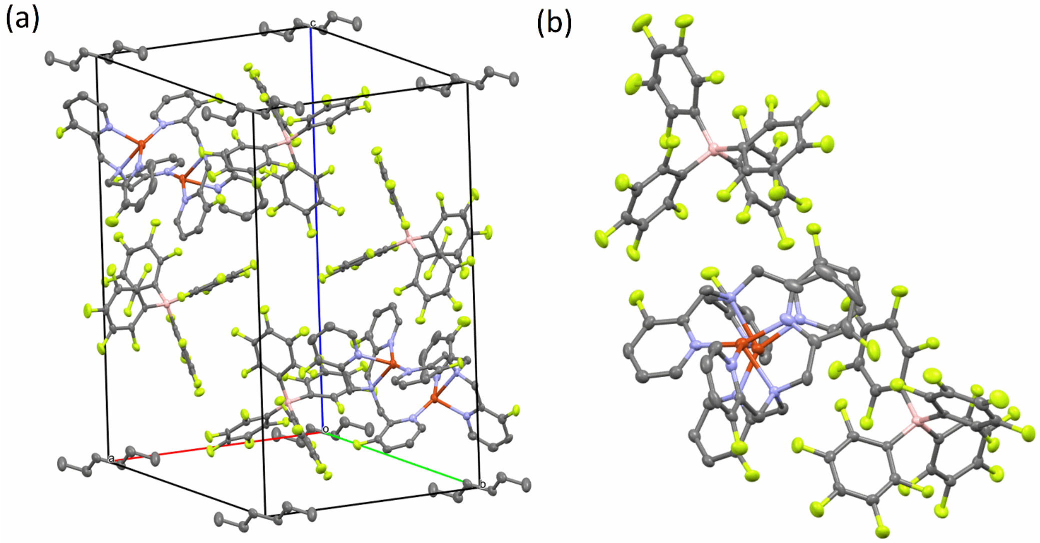

Figure S46. Diagram illustrating the (a) molecular packing of **[(F$_2$tmpa)$_2$Cu$^I_2$][B(C$_6$F$_5$)$_4$]$_2$** and (b) the corresponding displacement ellipsoid plot with 50% probability level at 100(2) K. All hydrogens and disordered solvent molecules have been omitted for clarity. The occupancy of the two F atoms attached pyridyl arms in the complex is 0.66 due to the random distribution of the three pyridyl arms during crystallization.

References

1. R. Li, F. S. T. Khan, M. Tapia and S. Hematian, *J. Coord. Chem*, 2022, **75**, 1617-1635.
2. S. Hematian, M. A. Siegler and K. D. Karlin, *J. Am. Chem. Soc.*, 2012, **134**, 18912-18915.
3. F. S. T. Khan, A. L. Waldbusser, M. C. Carrasco, H. Pourhadi and S. Hematian, *Dalton. Trans.*, 2021, **50**, 7433-7455.
4. R. S. Nicholson, *Anal. Chem.*, 1965, **37**, 1351-1355.
5. W. Linert, Y. Fukuda and A. Camard, *Coord. Chem. Rev.*, 2001, **218**, 113-152.
6. Y. Chernyak, *J. Chem. Eng. Data*, 2006, **51**, 416-418.
7. F. Barrière and W. E. Geiger, *J. Am. Chem. Soc.*, 2006, **128**, 3980-3989.
8. V. Gutmann, *Coord. Chem. Rev.*, 1976, **18**, 225-255.
9. N. G. Connelly and W. E. Geiger, *Chemical Reviews*, 1996, **96**, 877-910.
10. J. S. Chen and R. B. Shirts, *J. Phys. Chem.*, 1985, **89**, 1643-1646.
11. G. Sheldrick, *Institute for Inorganic Chemistry. University of Göttingen, Göttingen, Germany*, 2013.
12. G. M. Sheldrick, *Acta Crystallogr. C Struct. Chem.*, 2015, **71**, 3-8.
13. L. Yang, D. R. Powell and R. P. Houser, *Dalton. Trans.*, 2007, DOI: 10.1039/B617136B, 955-964.
14. A. Okuniewski, D. Rosiak, J. Chojnacki and B. Becker, *Polyhedron*, 2015, **90**, 47-57.
